# Effects of gelsemine on oxidative stress and DNA damage responses of *Tetrahymena thermophila*

**DOI:** 10.7717/peerj.6093

**Published:** 2018-12-10

**Authors:** Qiao Ye, Yongyong Feng, Zhenlu Wang, Wenzhao Jiang, Yuexin Qu, Chaonan Zhang, Aiguo Zhou, Shaolin Xie, Jixing Zou

**Affiliations:** 1 Healthy Aquaculture Laboratory, College of Marine Sciences, South China Agricultural University, Guangzhou, Guangdong, China; 2 Joint Laboratory of Guangdong Province and Hong Kong Region on Marine Bioresource Conservation and Exploitation, College of Marine Sciences, South China Agricultural University, Guangzhou, Guangdong, China

**Keywords:** *T. thermophila*, Gelsemine, Gene expression, Oxidative stress, DNA damage

## Abstract

Gelsemine is an important toxic substance extracted from *Gelsemium elegans*, which has a lot of biological functions in cells and organisms, but its toxicity has been rarely reported in *Tetrahymena thermophila*. In this study, we used the protozoan *T. thermophila* as an experimental model to investigate the potential toxicity-induced mechanism of gelsemine in the unicellular eukaryote. Our results clearly showed gelsemine inhibited *T. thermophila* growth in a dose-dependent manner. This exposure also resulted in oxidative stress on *T. thermophila* cells and antioxidant enzyme levels were significantly altered at high gelsemine levels (*p* < 0.05). Gelsemine produced a slight apoptotic effect at the highest (0.8 mg/mL) gelsemine level used here (*p* < 0.05). Furthermore, the toxin-induced DNA damage in a dose-dependent manner. The ultrastructural analysis also revealed mitophagic vacuoles at 0.4 and 0.8 mg/mL levels of gelsemine exposure. Moreover, expressions of oxidative stress-related and MAP kinase genes were significantly changed after exposure to 0.8 mg/mL level of gelsemine (*p* < 0.05). Altogether, our results clearly show that gelsemine from *G. elegans* can inhibit the growth via inducing oxidative stress and DNA damage in *T. thermophila* cells.

## Introduction

*Gelsemium elegans* Benth is a famous medicinal plants native to Southeast Asia, particularly in China, which has been long used for centuries as a traditional Chinese folk medicine, in the treatment of inflammation, anxiety and neuralgia in spite of their toxicity (Liu et al., 2011a, 2013). Although more than 190 chemicals have been identified in *G. elegans*, the primary bioactive components in it are alkaloids, which extracted from the plant have been found possessing various biological effects (Jin et al., 2014). Various alkaloids have similar structures, but they are diverse in pharmacological actions and toxicities. Gelsemine is the only common alkaloid between *G. elegans* and *G. sempervirens* while koumine and gelsenicine are unique to *G. elegans* (Wu et al., 2015). Gelsemine is highly toxic and although its chemical structure and stereochemistry have been defined (Lai & Chan, 2009), its biological effects are poorly described. There is only one report that gelsemine directly modulated recombinant and native glycine receptors in vertebrate (Lara et al., 2016). There is increased interest in gelsemine since it can alter many biological activities in vertebrates, especially in mammals. However, the effects of gelsemine on microorganisms have not been investigated.

Some diseases caused by the unicellular organisms including *Echinococcus multilocularis*, *Ichthyophthirius multifiliis* and *Vorticella*, have brought a serious impact on aquaculture, livestock and humans risks (Moyses et al., 2015; Bansal et al., 2018). We here chose the widely distributed ciliated protozoa *Tetrahymena thermophila*, a representative for the study on unicellular organisms (Lynn & Doerder, 2012), as a model system to study the effects of gelsemine on unicellular microorganisms. Recently, the genome of *T. thermophila* has been assembled and annotated, laying a good foundation on downstream studies of functional genomics (Eisen et al., 2006; Stover et al., 2006). As an important unicellular model organism, *T. thermophila* can be easily cultured and it has a short generation time in a small volume of culture medium. Due to its convenience for cultivation under laboratory conditions and sensitivity to chemical exposure, *T. thermophila* has been commonly selected as a standard living model to evaluate the effects of chemicals and thereby explore molecular mechanisms. A large number of previous studies have indicated *T. thermophila* as an effective model organism, being a pharmacological tool in different bioassay techniques to detect toxicants (Sauvant, Pepin & Piccinni, 1999; Eisen et al., 2006).

The effects of gelsemine on *T. thermophila* have not been studied so far. To be specific, we evaluated the effects of gelsemine on oxidative damage, apoptosis and DNA damage in *T. thermophila* cells, and the expression pattern of oxidative stress-related genes in *T. thermophila* with the administration of gelsemine. Therefore, the effects of gelsemine on *T. thermophila* cells and the molecular mechanisms underlying that were comprehensively explored and discussed.

## Materials and Methods

### Species and processing of gelsemine

The strain B2086 of *T. thermophila* was obtained from Professor Miao (Institute of Hydrobiology, Chinese Academy of Sciences, Wuhan, China). Prior to the experiments, the *T. thermophila* were cultured at 30 °C with shaking at 135 rpm in super proteose peptone (SPP) medium, which consists of 2% proteose peptone, 0.1% yeast extract, 0.2% glucose and 0.003% ferric citrate (Chang, Feng & Miao, 2011). Culture medium was sterilized in high-pressure steam at 120 °C for 30 min before use. Gelsemine (purity: 99.28%) was purchased from Chengdu Mansite Bio-Technology Co., Ltd (Chengdu, China). The stock solutions of gelsemine decompose into dimethyl sulfoxide (DMSO) under 60 °C. Gelsemine was exposed to ultraviolet germicidal irradiation (103 μW/cm^2^, used UIT-250 UV intensity detector, USHIO, Japan) for 1 h prior to the experiment to eliminate potential microbial contamination.

### Growth curves and cells viabilities

In order to obtain the cell densities of *T. thermophila* treated with different concentrations of gelsemine, growth curves were produced. After 24 h culture, the logarithmic period cells were inoculated into 10 mL SPP media in 50 mL centrifuge tube with 6,250 cells/mL seeding density, and the medium was mixed with different gelsemine contents, thereby obtaining final gelsemine concentrations with 0, 0.05, 0.1, 0.2, 0.4 and 0.8 mg/mL, respectively. Each concentration was conducted in triplicate. zero mg/mL concentration of gelsemine was set as a blank control, and 0.1% DMSO, the vehicle for gelsemine addition, served as a negative control (NC). *T. thermophila* cells were cultured for 72 h at 30 °C with shaking at 135 rpm. Cell densities were determined using Countstar Automated Cell Counter (Countstar, Shanghai, China) during the culture period. Cell viability was determined by counting the relative cell number in the presence of gelsemine for 24 h (Cell counting kit-8 used, Nanjing, Jiancheng, China) according to the manufacturer’s instructions. Briefly, the cell suspension (1 × 10^4^ cells/L) was added into the 96 hole plate, and add 10 μL Cell counting kit-8 (CCK-8) solution per hole, then the plates were incubated for 3 h in incubators, and the absorbance at 450 nm was measured by enzyme labelling apparatus. CCK-8 allows sensitive colorimetric assays for the determination of cell viability in cell proliferation and cytotoxicity assays. The highly water-soluble tetrazolium salt, WST-8, is reduced by dehydrogenase activities in cells to give a yellow-color formazan dye, which is soluble in the tissue culture media. The amount of the formazan dye, generated by the activities of dehydrogenases in cells, is directly proportional to the number of living cells. The half-maximal effective concentration (EC_50_) of gelsemine was calculated.

### Cellular enzymic activity analysis

*Tetrahymena thermophila* culture was exposed to various levels of gelsemine, and cells were harvested by centrifugation of 3,500 rpm for 15 min and washed twice with cold phosphate buffer solution (PBS, pH 7.4) at room temperature, and then suspended in PBS. The supernatant was collected to assay the content of malondialdehyde (MDA). A malonaldehyde detection kit (Nanjing Jiancheng Bioengineering Institute, Nanjing, China) was used for MDA determination. Simply, thiobarbituric acid reacted with MDA to form red products, and the absorbance was measured at 532 nm. The MDA concentration was expressed as nanomoles of MDA per milligram of protein (Rongzhu et al., 2009). The activities of superoxide dismutase (SOD), catalase (CAT), peroxidase (POD) and glutathione peroxidase (GSH-PX) were measured using a commercial reagent kit (Nanjing Jiancheng Bioengineering Institute, Nanjing, China) spectrophotometrically. The activities of SOD, CAT and POD were expressed as units per milligram protein. The activity of SOD was assayed at 550 nm using the xanthine and xanthine oxidase system. One unit (U) of SOD activity was defined as the amount of enzyme causing 50% inhibition of xanthine and xanthine oxidase reaction system. The samples were treated with the excess of H_2_O_2_ at 37 °C, and the absorbance of remaining H_2_O_2_ was measured at 405 nm to determine the activity of CAT. The samples were treated with H_2_O_2_ at 37 °C for 30 min. POD activity was determined by detection of changes in the absorbance at 420 nm. The samples were treated with glutathione (GSH, 20 μmol/L) at 37 °C for 5 min, GSH-PX activity was determined by detection of changes in the absorbance at 412 nm. The content of total protein was measured by coomassie blue staining, using bovine serum albumin (Solarbio, China) as a standard (Ma et al., 2015). Among the 14 known caspases, Caspases 3, 8 and 9 are most closely related to apoptosis and play an important role in apoptosis. The Caspases 3, 8 and 9 were measured using the assay kits provided by Nanjing Jiancheng Biotechnique Institute (Nanjing, China) in line with the manufacturer’s protocol. Caspase sequence-specific polypeptides were coupled to chromophore groups. When the substrate was cleaved by a specific caspase, the chromophore groups were free, and the absorbance value could be determined by spectrophotometer (λ = 405 nm).

### Apoptosis analysis using flow cytometric

The *T. thermophila* cells were separately exposed to various levels of gelsemine for 24 h at 30 °C with constant shaking (135 rpm) at 6,250 cells/mL inoculation concentration. Afterward, cells were collected by centrifugation at 3,500 rpm for 15 min and washed twice with cold PBS (pH 7.4) at room temperature. The fluorescein isothiocyanate-Annexin V was added to *T. thermophila* cells, mixed thoroughly and incubated for 10 min in an ice bath in the dark. The *T. thermophila* cells were then washed twice with PBS and analyzed on a flow cytometer. A total of 10,000 cells were obtained and examined per sample using a Beckman–Coulter EPICS XL flow cytometer (Brea, CA, USA). Data produced above was further analyzed using the Cell Quest software.

### Comet assay

The comet assay was used to evaluate the DNA damage of cells in different concentrations of gelsemine exposure. *T. thermophila* cells were collected by centrifugation at 3,500 rpm for 15 min and washed twice in PBS. The assay was conducted using a commercial kit (Comet Assay Kit, Nanjing, Jiancheng, China). In brief, cell suspensions were mixed with low melting point agarose (0.8%) and poured on glass slides. The slides were then immersed in a cold lysis solution at 4 °C for 2 h followed by electrophoresis at 4 °C for 30 min. The slides were stained with EB (ethidium bromide) and photographed using a fluorescence microscope. The images were analyzed using Comet Assay Software (CASP 1.2.3 beta 1) (Konca et al., 2003). Olive tail moment (OTM) was used for assessing DNA damage. The experiments were performed in three biological repetitions for each.

### Transmission electron microscopy imaging

Transmission electron microscopy (TEM) was performed by a commercial company (Wuhan Servicebio Technology, Guangzhou, China) using a previously described procedure (Li et al., 2015a). Briefly, *T. thermophila* cells were fixed in sodium cacodylate buffer containing 2.5% glutaraldehyde at room temperature for 1 h and then incubated in 2% aqueous osmium tetroxide for 45 min at 4 °C. Then the fixed cells were immersed in 1% uranyl acetate for 1 h, dehydrated in an acetone series and embedded in epoxy resins, thereby obtaining the stained ultrathin sections. Hitachi HT7700 TEM (Hitachi, Chiyoda, Tokyo, Japan) was used for imaging of *T. thermophila* ultrastructure.

### Gene expression analysis

Total RNA was isolated from *T. thermophila* using an RNAiso Plus kit (Takara, Dalian, China) according to the manufacturer’s protocol. RNA integrity was evaluated by 2% agarose gel electrophoresis and then reverse transcribed (RT) using a commercial kit (Takara, Dalian, China) according to the manufacturer’s instructions. The cDNA was used for quantitative real-time PCR analysis (qRT-PCR). Gene sequences for *MTT2/4* (metallothionein 2, NW_002476246), *CYP1* (cytochrome P450, family 1, NW_002476416), *HSP70* (heat shock protein 70, NW_002476481), *MPK1* (mitogen-activated protein kinase 1, NW_002476155), *MPK3* (mitogen-activated protein kinase 3, NW_002476348), *ATG7* (autophagy 7, NW_002476353) genes and 18S rRNA (HE820726.1) were all retrieved from GenBank (https://www.ncbi.nlm.nih.gov/genbank/). Owing to the high homology of *MTT2* and *MTT4* nucleotides (De Francisco et al., 2017), we only can to analyze the expression of both genes as a set (*MTT2/4*) by RT-qPCR. PCR primers were designed using Primer Premier 5.0 software (Table S1). Relative gene expression was calculated and normalized with the quantity of 18S rRNA present in each sample. The qRT-PCR was performed using SYBR Premix Ex Taq II (Takara, Dalian, China) using a BioRad CFX96 Real-Time PCR Detection System (Bio-Rad, Hercules, CA, USA). The cycling conditions were 95 °C for 2 min, followed by 39 cycles of 95 °C for 15 s, 60 °C for 30 s, 72 °C for 30 s and a final step of 72 °C for 7 min. Real-time PCR data were analyzed using the 2^–ΔΔCt^ method.

### Statistical analysis

Data for cell viability assays, DNA damage and cellular enzymic activities were analyzed using the SPSS statistical package version 19.0 and analysis of variance (ANOVA). Real-time PCR data were analyzed using the 2^–ΔΔCt^ method. Differences between groups were analyzed using the SPSS (ANOVA, least significant difference (LSD)), with *p* < 0.05 considered to be statistically significant. The residuals of the models were analyzed, and the residuals coincide with the normal distribution. The homogeneity of variances was analyzed using Bartlett test. All values were expressed as the mean ± standard deviation and all experiments were performed with three biological repetitions for each.

EC_50_ values were calculated with their 95% confidence interval from the data obtained using the software package GraphPad Prism 5.0. EC_50_ calculations were fitted to a sigmoidal four-parameter dose-response model using the equation:

$$y = {{b + (a-b)} \over {(1 + {{10}^{({\rm{LogE}}{{\rm{C}}_{50}}-x)}}h)}}$$

where *y* is the response, *b* is the response minimum, *a* is the response maximum, *h* is the shape parameter and *x* is the logarithm of the inhibitor concentration.

## Results

### Effect of growth inhibition in *T. thermophila*

**Figure 1:**
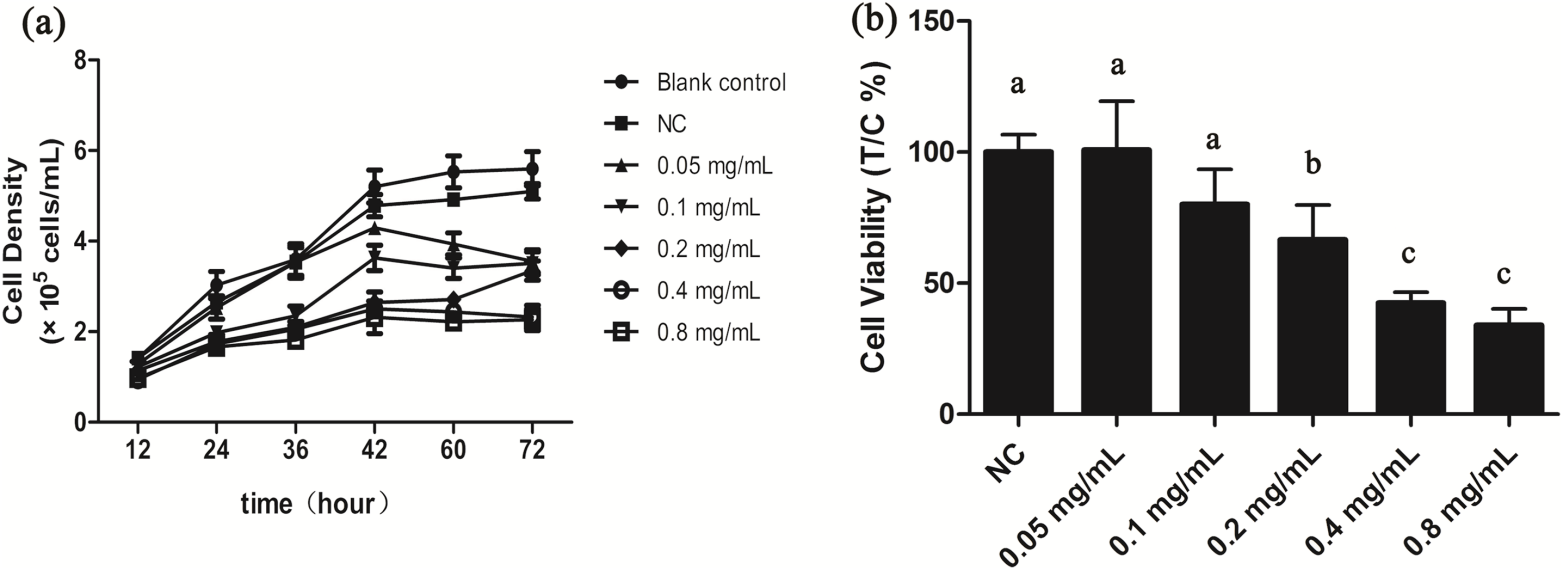
Gelsemine affects *T. thermophila* proliferation. (A) Growth curves of *T. thermophila* based on numbers of cells exposed to various concentrations of gelsemine for 72 h. (B) Cell viabilities of *T. thermophila* exposed to gelsemine for 24 h. Data are expressed as mean ± SD for *n* = 3. Values with no common superscript differ significantly (*p* < 0.05) or are highly significant (*p* < 0.01). Blank control, no added drug; Negative control (NC), vehicle only (0.1% DMSO).

We investigated potential growth effects of gelsemine on *T. thermophila* by measuring cell densities in the presence and absence of the gelsemine. Growth was inhibited by gelsemine in a dose-dependent manner, and its extent of inhibition increased with increasing gelsemine concentration (Fig. 1; Table S2). Moreover, all the treatment group reached the growth plateau stage after a 42-h culture, earlier than the controls (Fig. 1A). Cell viability was also adversely affected by gelsemine, and this was especially apparent in the concentrations of 0.2, 0.4 and 0.8 mg/mL treatment groups (*p* < 0.05), which were also dose-dependent. The EC_50_ at 24 h was calculated at 0.48 mg/mL using this data (Fig. 1B).

### Effect of oxidative stress in *T. thermophila*

**Figure 2:**
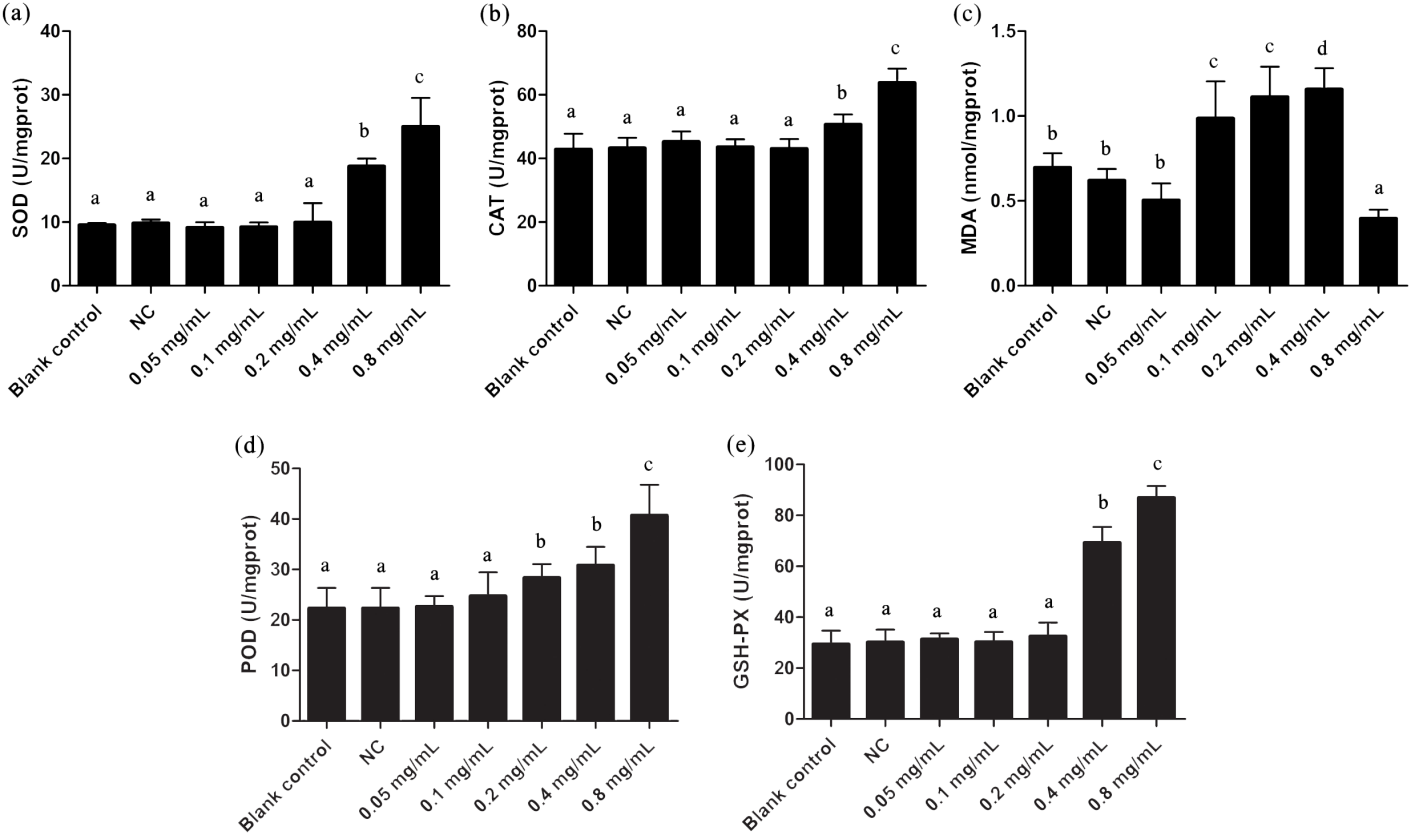
Antioxidant enzyme activities of *T. thermophila* after gelsemine exposure. (A) SOD, (B) CAT, (C) MDA, (D) POD, (E) the sum of GSH-PX. Data are expressed as mean ± SD for *n* = 3. Values with no common superscript differ significantly (*p* < 0.05) or are highly significant (*p* < 0.01). Blank control, no added drug; Negative control (NC), vehicle only (0.1% DMSO).

**Figure 3:**
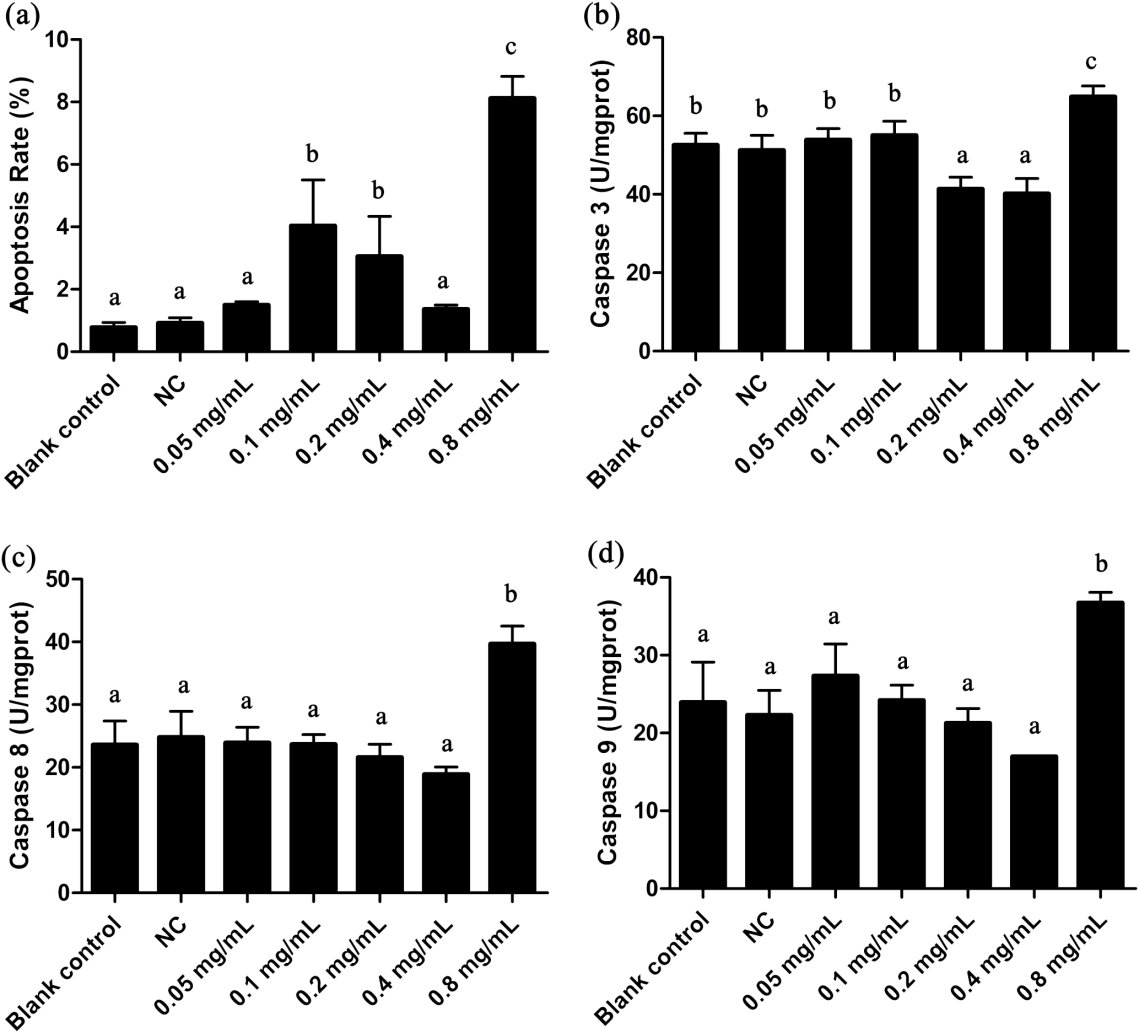
Apoptosis rate and Caspases 3, 8, 9 activities of *T. thermophila* after exposure to gelsemine at various concentrations. (A) Apoptosis rate, (B) Caspase 3 activity, (C) Caspase 8 activity, (D) Caspase 9 activity. Data are expressed as mean ± SD for *n* = 3. Values with no common superscript differ significantly (*p* < 0.05) or are highly significant (*p* < 0.01). Blank control, no added drug; Negative control (NC), vehicle only (0.1% DMSO).

The oxidative stress effects of gelsemine were evaluated by measuring the levels of antioxidant enzymes and MDA content. SOD, CAT, POD and GSH-PX enzyme activities were all elevated significantly in cells with the administration of both 0.4 and 0.8 mg/mL gelsemine (*p* < 0.05). There were no significant changes in other treatment groups (Fig. 2). Additionally, MDA content was significantly decreased at 0.8 mg/mL gelsemine (*p* < 0.01) and significantly increased at 0.1, 02 and 0.4 mg/mL (*p* < 0.01) (Fig. 3C). This data indicates that gelsemine produces significant alterations in cellular redox status at high concentrations and that the compound causes oxidative stress in *T. thermophila*.

### Effect of apoptosis in *T. thermophila*

The apoptotic state is an important indicator to evaluate the cell states, and it can be well identified by flow cytometric. As shown in Fig. 3A and Fig. S1G, the apoptosis rate in 0.8 mg/mL treatment group was under approximately 8.8%, indicating a certain extent of apoptosis. Caspases 3, 8 and 9 are three important enzymes closely correlative with apoptosis. In *T. thermophila*, we here observed that the activities of Caspases 3, 8 and 9 exposed to 0.8 mg/mL of gelsemine were all significantly increased (*p* < 0.01) compared to that in the NC group (Fig. 3). The above results collectively indicate that a relatively high concentration of gelsemine may induce the apoptosis in *T. thermophila*.

### Effects of DNA damage in *T. thermophila*

**Figure 4:**
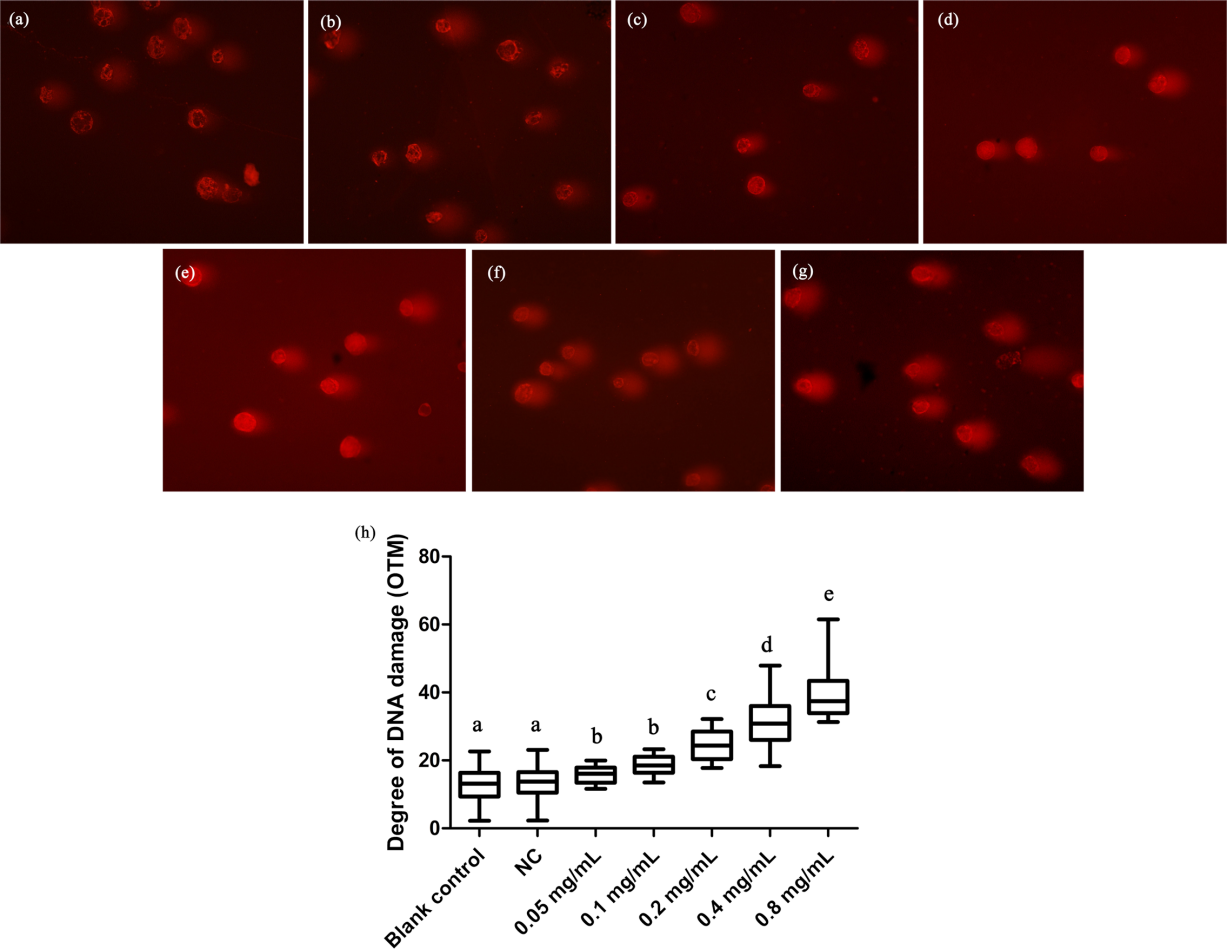
Comet assay to detect DNA damage in *T. thermophila* cells exposed to gelsemine. (A) Blank control, (B) Negative control (NC), vehicle only (0.1% DMSO) (C) 0.05 mg/mL, (D) 0.1 mg/mL, (E) 0.2 mg/mL, (F) 0.4 mg/mL, (G) 0.8 mg/mL, (H) DNA damage in *T. thermophila* exposed to increasing concentrations of gelsemine for 24 h. Data are expressed as mean ± SD for *n* = 3. Values with no common superscript differ significantly (*p* < 0.05) or are highly significant (*p* < 0.01). Blank control, no added drug; Negative control (NC), vehicle only (0.1% DMSO).

We here employed comet assay to reveal the effects of DNA damage in *T. thermophila*. We found that all the concentrations of gelsemine caused DNA damage in a dose-dependent manner (Fig. 4). The OTM values were significantly increased at all gelsemine treatment groups compared to the NC control group (*p* < 0.05). Even at low concentrations (0.05 or 0.1 mg/mL), we could find evidence of DNA damage (Fig. 4H). In addition, the extent of damage was greater (40–60%) at 0.4 and 0.8 mg/mL levels (Table S3). The results presented above clearly indicate that gelsemine can cause DNA damage in *T. thermophila*.

### Ultrastructural analysis

**Figure 5:**
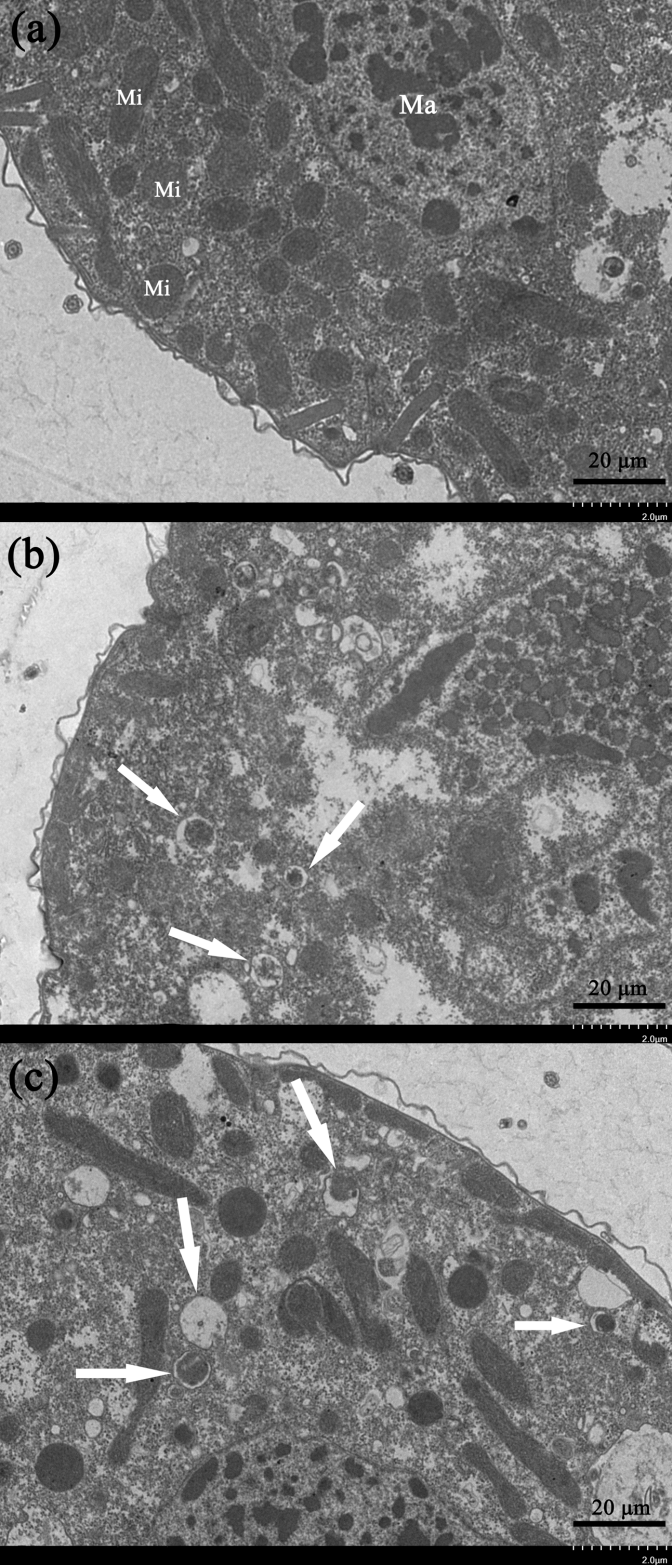
Ultrastructural analysis by TEM of *T. thermophila* cells exposed to increasing concentrations of gelsemine for 24 h. (A) NC control group showing a normal mitochondrion. Macronucleus (Ma), Mitochondrion (Mi). (B) A total of 0.4 mg/mL treatment group showing preliminary evidence of mitophagic vacuoles (arrows). (C) A total of 0.8 mg/mL treatment group showing overt mitophagic vacuoles (arrows).

Transmission electron microscopy is an effective way to observe ultrastructural changes in the cells. We here carried out the TEM experiments to investigate the potential ultrastructural changes of cells brought by the gelsemine treatment. Consequently, a series of obvious changes in the cellular ultrastructure of *T. thermophila* exposed to various concentration of gelsemine could be observed, along with signs of mitophagic vacuoles at the higher treatment levels (0.4 and 0.8 mg/mL) identified (Figs. 5B and 5C, white arrows). These results indicate that the mitophagic vacuoles presented here much more likely correlates with the cell death induced by gelsemine treatment.

### Expression of stress-related genes in *T. thermophila*

**Figure 6:**
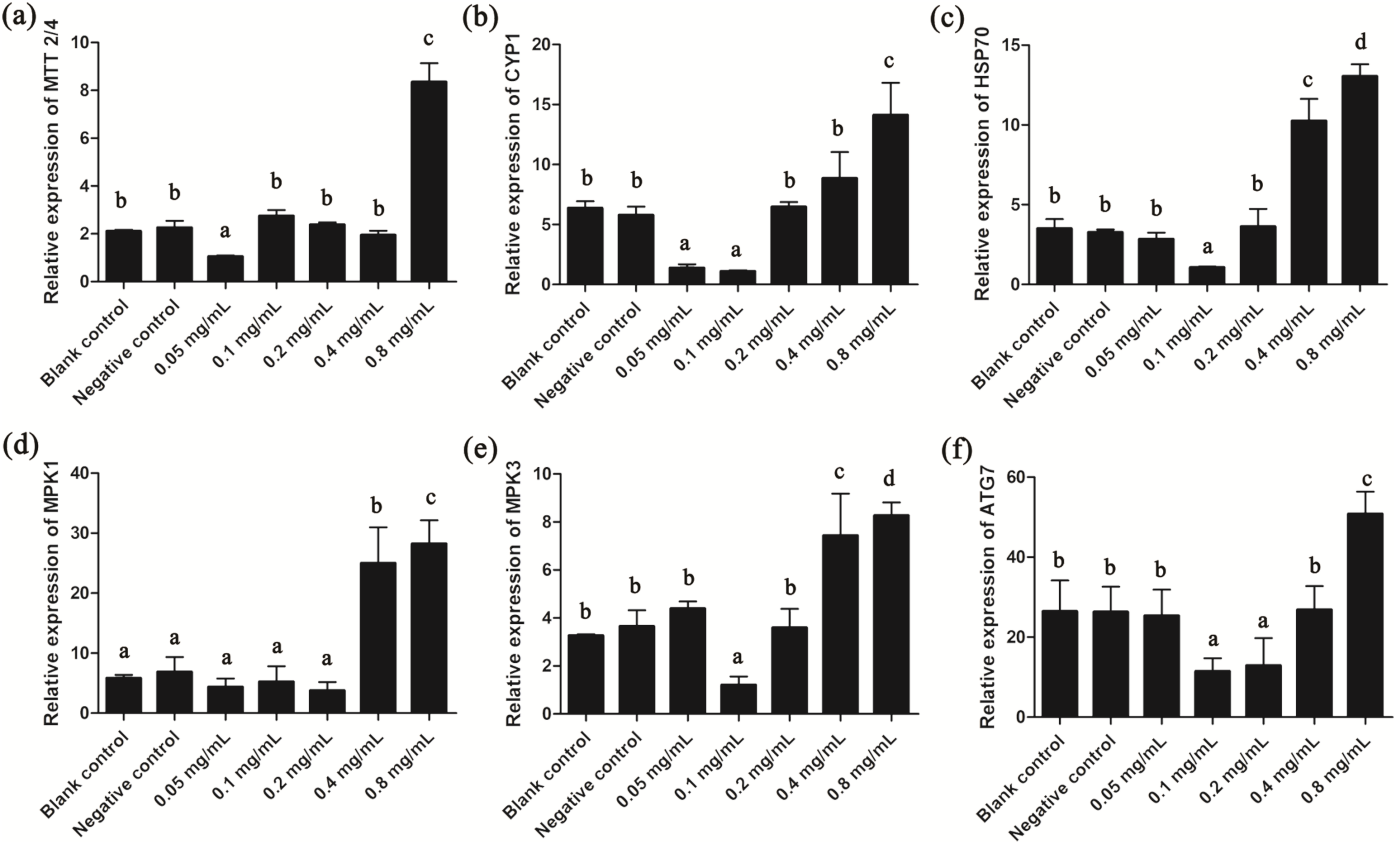
Relative expression levels of *T. thermophila* stress-related genes after gelsemine treatment for 24 h. Expression levels were measured using qRT-PCR and normalized against 18S rRNA. (A) MTT2/4, (B) CYP1, (C) HSP70, (D) MPK1, (E) MPK3, (F) ATG7. Data are expressed as mean ± SD for *n* = 3. Values with no common superscript differ significantly (*p* < 0.05) or are highly significant (*p* < 0.01). Blank control, no added drug; Negative control (NC), vehicle only (0.1% DMSO).

Since gelsemine could cause cellular stress on *T. thermophila* cells, we then measured the expression patterns of several stress-related genes to draw a more complete picture of the biological actions of this chemical. Results showed that the expression of *MTT2/4, CYP1* and *HSP70* were all significantly reduced at low gelsemine levels and significantly increased (*p* < 0.01) at 0.8 mg/mL (Figs. 6A–6C). These results were consistent with our antioxidant enzymes results since these three genes are induced during the process of oxidative stress. In addition, the expression levels of *MPK1* and *MPK3* were both significantly increased at 0.4 and 0.8 mg/mL gelsemine (*p* < 0.05) (Figs. 6D and 6E), indicated a potential activation of MAP kinase pathway triggered by gelsemine treatment in *T. thermophila*. We also found a significant increase in *ATG7* expression at 0.8 mg/mL, which correlated with autophagy-related processes (Fig. 6F).

## Discussion

Some pathogenetic eukaryotic microorganism, such as *E. multilocularis*, *I. multifiliis* and *Vorticella* caused potential aquatic ecological and human health risks (De Padua et al., 2013; Moyses et al., 2015; Bansal et al., 2018). *T. thermophila* is an excellent research model of these pathogenic eukaryotic microorganisms. Therefore, evaluation of the toxicity of gelsemine in *T. thermophila* has an important reference value for its further potential utilization in the disease control for livestock in general and humans. Toxicity and molecular mechanisms of biological responses to gelsemine have been previously examined in vertebrates. To the best of our knowledge, the current study is the first to examine the effects of gelsemine on a microorganism. Toxicities of hazardous substances are routinely determined by analysis of dynamic growth curves of *T. thermophila.* In our study, *T. thermophila* cell densities were inversely proportional to the gelsemine concentration in the culture medium. Cell viability also decreased in a dose-dependent manner as the gelsemine levels were increased. In general, the results of cell viability assays were consistent with that of the growth inhibition tests. The results described above showed that gelsemine can inhibit the growth of *T. thermophila.*

Beyond normal physiological roles, excessive reactive oxygen species (ROS) production can occur in response to toxin exposure, resulting in local oxidative stress (Deavall et al., 2012). Cells usually prevent or limit intracellular damage and ameliorate the harmful effects of ROS using antioxidant enzymes such as SOD, POD, CAT and GSH-PX (Deavall et al., 2012). The MDA produced by lipid peroxidation is a biomarker that reflects overall levels of oxidative stress (Liu et al., 2011b). Some chemicals, such as myclobutanil and cyproconazole caused notable changes in enzyme activities lead to oxidative stress in *T. thermophila* (Huang et al., 2016). In the present study, SOD, POD, GSH-PX and CAT in *T. thermophila* were significantly increased (*p* < 0.05) in the 0.4 and 0.8 mg/mL treatment groups. This indicates that gelsemine stimulates the production of ROS, thereby leading to oxidative stress. However, MDA content was significantly decreased at 0.8 mg/mL, and MDA was a common index used for membrane lipid peroxidation (Li et al., 2018). It means the gelsemine may first affect the oxidation of lipid in the cell membrane. Overall, activities of antioxidant enzymes were highly elevated indicating that gelsemine induces oxidative stress via ROS production.

Oxidative stress can also stimulate apoptosis (Wu et al., 2013; Yu et al., 2015). High melamine concentrations damaged the *T. thermophila* genome to a certain extent and induced apoptosis in the organism (Li et al., 2015b). We here found an obvious gelsemine-induced apoptosis in *T. thermophila*. The cellular apoptosis rates were elevated in the 0.8 mg/mL treatment group. In addition, Caspases 3, 8 and 9 were all significantly increased in different concentrations of gelsemine compared with that in controls. It has been shown that ROS can induce DNA damage. Under severe oxidative stress, high levels of DNA damage cause apoptosis in the damaged cells (Deavall et al., 2012; Belfield et al., 2014). The triclosan and triclocarban compound can lead to statistically significant DNA damage in *T. thermophila* (Gao et al., 2015). Thus, we wanted to determine whether gelsemine would produce the DNA damage effects in *T. thermophila*. Our results indicated that gelsemine induced DNA damage in a dose-dependent manner. High gelsemine level (0.8 mg/mL) induced serious DNA damage while antioxidant enzymes were also significantly changed. Therefore, DNA damage may be associated with oxidative stress in *T. thermophila*. Oxidative stress has been implicated in various cells disorders and may be a major cause of cell death. For example, oxidative stress induced mitophagic vacuoles resulting in mitochondria-dependent autophagy (Eid et al., 2016). We found that mitophagic vacuoles occurred with gelsemine exposure at 0.4 and 0.8 mg/mL. This correlated with that of the significant changes in all the antioxidant enzymes we measured. This also agrees with mitochondria damage, and we found that was the result of gelsemine-induced oxidative stress, indicating mitochondria as targets of gelsemine toxicity.

The *MTT2/4* expression is induced by oxidative stress and toxins (Diaz et al., 2007; Figueira et al., 2012). *CYP1* gene participates both in toxin metabolism and oxidative stress (Rao et al., 2016). For instance, *CYP1* is induced by oxidative stress in the lungs and livers of rats exposed to incense smoke (Hussain et al., 2014). *HSP70* has antioxidant functions and can protect cells from stress. For example, *HSP70* is over-expressed in the preeclamptic placenta and *HSP70* protects human neuroblastoma cells from apoptosis and oxidative stress (Padmini, Uthra & Lavanya, 2011; Yurinskaya et al., 2015). In our study, we found that *MTT2/4*, *CYP1* and *HSP70* were all increased significantly at 0.8 mg/mL gelsemine and this correlated with increased levels of oxidative stress. Taken together, these findings indicate that *MTT2/4*, *CYP1* and *HSP70* are also closely associated with the oxidative stress response to gelsemine in *T. thermophila.*

Protein kinases regulate cellular signals controlling growth, proliferation and survival in response to stress. Multiple kinase signaling pathways are affected by oxidative stress and kinase activation is critical in detecting oxidative stress (Ryter et al., 2007; Zhong et al., 2014). *MPK1* and *MPK3* genes are two important genes in MAP kinase signaling and can be activated by oxidative stress (Patel et al., 2011; Perez-Salamo et al., 2014). In our study, the expression levels of *MPK1* and *MPK3* increased significantly at the 0.4 and 0.8 mg/mL gelsemine implicating that gelsemine may affect the *T. thermophila* through the MAPK pathway. In addition, *ATG7* as an important regulator of autophagy signal transduction and oxidative stress (Zhuo et al., 2013; Desai et al., 2013; Gonzalez et al., 2014), was also found to be significantly increased in the 0.8 mg/mL treatment group, which was consistent with our oxidative stress and electron microscopy experiments.

## Conclusion

In summary, we here conducted a comprehensive investigation of potential gelsemine toxicity to *T. thermophila.* We found that gelsemine could exert oxidative stress, apoptosis, DNA damage and mitophagic vacuoles on *T. thermophila*, possibly via activating the MAPK pathway. In conclusion, our study clearly indicates that gelsemine has an antagonistic effect on eukaryotic single cell microorganisms. We believe the present study will aid in understanding the toxicity mechanisms of gelsemine on *T. thermophila*, as well as provide a theoretical basis for its possible practical utilization in pathogenetic eukaryotic microorganism control.

## Supplemental Information

10.7717/peerj.6093/supp-1 Supplemental Information 1 Supplementary materials of the manuscript.

## Additional Information and Declarations

### Data Availability

The following information was supplied regarding data availability: Raw data is available in the Supplemental File.

## References

[ref-1] Bansal N, Vij V, Rastogi M, Wadhawan M, Kumar A (2018). A report on three patients with *Echinococcus multilocularis*: lessons learned. Indian Journal of Gastroenterology.

[ref-2] Belfield C, Queenan C, Rao H, Kitamura K, Walworth NC (2014). The oxidative stress responsive transcription factor Pap1 confers DNA damage resistance on checkpoint-deficient fission yeast cells. PLOS ONE.

[ref-3] Chang Y, Feng L, Miao W (2011). Toxicogenomic investigation of *Tetrahymena thermophila* exposed to dichlorodiphenyltrichloroethane (DDT), tributyltin (TBT), and 2,3,7,8-tetrachlorodibenzo-p-dioxin (TCDD). Science China Life Sciences.

[ref-4] De Francisco P, Martin-Gonzalez A, Turkewitz AP, Gutierrez JC (2017). Extreme metal adapted, knockout and knockdown strains reveal a coordinated gene expression among different *Tetrahymena thermophila* metallothionein isoforms. PLOS ONE.

[ref-5] De Padua SB, Ishikawa MM, Ventura AS, Jeronimo GT, Martins ML, Tavares LER (2013). Brazilian catfish parasitized by Epistylis sp. (Ciliophora, Epistylididae), with description of parasite intensity score. Parasitology Research.

[ref-6] Deavall DG, Martin EA, Horner JM, Roberts R (2012). Drug-induced oxidative stress and toxicity. Journal of Toxicology.

[ref-7] Desai S, Liu Z, Yao J, Patel N, Chen J, Wu Y, Ahn EE-Y, Fodstad O, Tan M (2013). Heat shock factor 1 (HSF1) controls chemoresistance and autophagy through transcriptional regulation of autophagy-related protein 7 (ATG7). Journal of Biological Chemistry.

[ref-8] Diaz S, Amaro F, Rico D, Campos V, Benitez L, Martin-Gonzalez A, Hamilton EP, Orias E, Gutierrez JC (2007). Tetrahymena metallothioneins fall into two discrete subfamilies. PLOS ONE.

[ref-9] Eid N, Ito Y, Horibe A, Otsuki Y (2016). Ethanol-induced mitophagy in liver is associated with activation of the PINK1-Parkin pathway triggered by oxidative DNA damage. Histology and Histopathology.

[ref-10] Eisen JA, Coyne RS, Wu M, Wu D, Thiagarajan M, Wortman JR, Badger JH, Ren Q, Amedeo P, Jones KM, Tallon LJ, Delcher AL, Salzberg SL, Silva JC, Haas BJ, Majoros WH, Farzad M, Carlton JM, Smith RJ, Garg J, Pearlman RE, Karrer KM, Sun L, Manning G, Elde NC, Turkewitz AP, Asai DJ, Wilkes DE, Wang Y, Cai H, Collins K, Stewart BA, Lee SR, Wilamowska K, Weinberg Z, Ruzzo WL, Wloga D, Gaertig J, Frankel J, Tsao CC, Gorovsky MA, Keeling PJ, Waller RF, Patron NJ, Cherry JM, Stover NA, Krieger CJ, Del TC, Ryder HF, Williamson SC, Barbeau RA, Hamilton EP, Orias E (2006). Macronuclear genome sequence of the ciliate *Tetrahymena thermophila*, a model eukaryote. PLOS Biology.

[ref-11] Figueira E, Branco D, Antunes SC, Goncalves F, Freitas R (2012). Are metallothioneins equally good biomarkers of metal and oxidative stress. Ecotoxicology and Environmental Safety.

[ref-12] Gao L, Yuan T, Cheng P, Bai Q, Zhou C, Ao J, Wang W, Zhang H (2015). Effects of triclosan and triclocarban on the growth inhibition, cell viability, genotoxicity and multixenobiotic resistance responses of *Tetrahymena thermophila*. Chemosphere.

[ref-13] Gonzalez Y, Aryal B, Chehab L, Rao VA (2014). Atg7- and Keap1-dependent autophagy protects breast cancer cell lines against mitoquinone-induced oxidative stress. Oncotarget.

[ref-14] Huang AG, Tu X, Liu L, Wang G-X, Ling F (2016). The oxidative stress response of myclobutanil and cyproconazole on *Tetrahymena thermophila*. Environmental Toxicology and Pharmacology.

[ref-15] Hussain T, Al-Attas OS, Al-Daghri NM, Mohammed AA, De Rosas E, Ibrahim S, Vinodson B, Ansari MG, El-Din KI (2014). Induction of CYP1A1, CYP1A2, CYP1B1, increased oxidative stress and inflammation in the lung and liver tissues of rats exposed to incense smoke. Molecular and Cellular Biochemistry.

[ref-16] Jin G-L, Su Y-P, Liu M, Xu Y, Yang J, Liao K-J, Yu C-X (2014). Medicinal plants of the genus *Gelsemium* (Gelsemiaceae, Gentianales)—a review of their phytochemistry, pharmacology, toxicology and traditional use. Journal of Ethnopharmacology.

[ref-17] Konca K, Lankoff A, Banasik A, Lisowska H, Kuszewski T, Gozdz S, Koza Z, Wojcik A (2003). A cross-platform public domain PC image-analysis program for the comet assay. Mutation Research/Genetic Toxicology and Environmental Mutagenesis.

[ref-18] Lai C-K, Chan Y-W (2009). Confirmation of gelsemium poisoning by targeted analysis of toxic gelsemium alkaloids in urine. Journal of Analytical Toxicology.

[ref-19] Lara CO, Murath P, Munoz B, Marileo AM, Martin LS, San MV, Burgos CF, Mariqueo TA, Aguayo LG, Fuentealba J, Godoy P, Guzman L, Yevenes GE (2016). Functional modulation of glycine receptors by the alkaloid gelsemine. British Journal of Pharmacology.

[ref-20] Li J, Giesy JP, Yu L, Li G, Liu C (2015a). Effects of tris(1,3-dichloro-2-propyl) phosphate (TDCPP) in *Tetrahymena thermophila*: targeting the ribosome. Scientific Reports.

[ref-21] Li W, Li H, Zhang J, Tian X (2015b). Effect of melamine toxicity on *Tetrahymena thermophila* proliferation and metallothionein expression. Food and Chemical Toxicology.

[ref-22] Li X-Y, Wei F, Gao J-S, Wang H-Y, Zhang Y-H (2018). Oxidative stress and hepatotoxicity of *Rana chensinensis* exposed to low doses of octylphenol. Environmental Toxicology and Pharmacology.

[ref-23] Liu M, Huang H-H, Yang J, Su Y-P, Lin H-W, Lin L-Q, Liao W-J, Yu C-X (2013). The active alkaloids of *Gelsemium elegans* Benth. Are potent anxiolytics. Psychopharmacology.

[ref-24] Liu M, Shen J, Liu H, Xu Y, Su YP, Yang J, Yu C-X (2011a). Gelsenicine from *Gelsemium elegans* attenuates neuropathic and inflammatory pain in mice. Biological and Pharmaceutical Bulletin.

[ref-25] Liu X-L, Xi Q-Y, Yang L, Li H-Y, Jiang Q-Y, Shu G, Wang S-B, Gao P, Zhu X-T, Zhang Y-L (2011b). The effect of dietary Panax ginseng polysaccharide extract on the immune responses in white shrimp, *Litopenaeus vannamei*. Fish and Shellfish Immunology.

[ref-26] Lynn DH, Doerder FP (2012). The life and times of Tetrahymena. Methods in Cell Biology.

[ref-27] Ma N, Li C, Dong X, Wang D, Xu Y (2015). Different effects of sodium chloride preincubation on cadmium tolerance of *Pichia kudriavzevii* and *Saccharomyces cerevisiae*. Journal of Basic Microbiology.

[ref-28] Moyses CRS, Spadacci-Morena DD, Xavier JG, Antonucci AM, Lallo MA (2015). Ectocommensal and ectoparasites in goldfish Carassius auratus (Linnaeus, 1758) in farmed in the State of Sao Paulo. Revista Brasileira de Parasitologia Veterinária.

[ref-29] Padmini E, Uthra V, Lavanya S (2011). HSP70 overexpression in response to ureaplasma urealyticum–mediated oxidative stress in preeclamptic placenta. Hypertension in Pregnancy.

[ref-30] Patel A, Chojnowski AN, Gaskill K, De Martini W, Goldberg RL, Siekierka JJ (2011). The role of a *Brugia malayi* p38 MAP kinase ortholog (Bm-MPK1) in parasite anti-oxidative stress responses. Molecular and Biochemical Parasitology.

[ref-31] Perez-Salamo I, Papdi C, Rigo G, Zsigmond L, Vilela B, Lumbreras V, Nagy I, Horvath B, Domoki M, Darula Z, Medzihradszky K, Bogre L, Koncz C, Szabados L (2014). The heat shock factor A4A confers salt tolerance and is regulated by oxidative stress and the mitogen-activated protein kinases MPK3 and MPK6. Plant Physiology.

[ref-32] Rao P, Ande A, Sinha N, Kumar A, Kumar S (2016). Effects of cigarette smoke condensate on oxidative stress, apoptotic cell death, and HIV replication in human monocytic cells. PLOS ONE.

[ref-33] Rongzhu L, Suhua W, Guangwei X, Chunlan R, Fangan H, Suxian C, Zhengxian Z, Qiuwei Z, Aschner M (2009). Effects of acrylonitrile on antioxidant status of different brain regions in rats. Neurochemistry International.

[ref-34] Ryter SW, Kim HP, Hoetzel A, Park JW, Nakahira K, Wang X, Choi AM (2007). Mechanisms of cell death in oxidative stress. Antioxidants & Redox Signaling.

[ref-35] Sauvant MP, Pepin D, Piccinni E (1999). *Tetrahymena pyriformis*: a tool for toxicological studies. A review. Chemosphere.

[ref-36] Stover NA, Krieger CJ, Binkley G, Dong Q, Fisk DG, Nash R, Sethuraman A, Weng S, Cherry JM (2006). Tetrahymena genome database (TGD): a new genomic resource for *Tetrahymena thermophila* research. Nucleic Acids Research.

[ref-37] Wu T, Chen G, Chen X, Wang Q, Wang G (2015). Anti-hyperlipidemic and anti-oxidative effects of gelsemine in high-fat-diet-fed rabbits. Cell Biochemistry and Biophysics.

[ref-38] Wu Y, Zhang X, Kang X, Li N, Wang R, Hu T, Xiang M, Wang X, Yuan W, Chen A, Meng D, Chen S (2013). Oxidative stress inhibits adhesion and transendothelial migration, and induces apoptosis and senescence of induced pluripotent stem cells. Clinical and Experimental Pharmacology and Physiology.

[ref-39] Yu JH, Cho SO, Lim JW, Kim N, Kim H (2015). Ataxia telangiectasia mutated inhibits oxidative stress-induced apoptosis by regulating heme oxygenase-1 expression. International Journal of Biochemistry and Cell Biology.

[ref-40] Yurinskaya MM, Mitkevich VA, Kozin SA, Evgen’Ev MB, Makarov AA, Vinokurov MG (2015). HSP70 protects human neuroblastoma cells from apoptosis and oxidative stress induced by amyloid peptide isoAsp7-Aβ(1–42). Cell Death and Disease.

[ref-41] Zhong W, Zhu H, Sheng F, Tian Y, Zhou J, Chen Y, Li S, Lin J (2014). Activation of the MAPK11/12/13/14 (p38 MAPK) pathway regulates the transcription of autophagy genes in response to oxidative stress induced by a novel copper complex in HeLa cells. Autophagy.

[ref-42] Zhuo C, Ji Y, Chen Z, Kitazato K, Xiang Y, Zhong M, Wang Q, Pei Y, Ju H, Wang Y (2013). Proteomics analysis of autophagy-deficient Atg7−/− MEFs reveals a close relationship between F-actin and autophagy. Biochemical and Biophysical Research Communications.

