# Supplementary material for: Effects of gelsemine on oxidative stress and DNA damage responses of Tetrahymena thermophila
Source: PeerJ. 2018 Dec 10;6:e6093. doi: 10.7717/peerj.6093 (PMC6292385; doi:10.7717/peerj.6093)
Supplement: Supplemental Information 1 [file peerj-06-6093-s001.doc]

**Effects of gelsemine on oxidative stress and DNA damage responses of Tetrahymena thermophila**

Qiao Ye^1, 2^ , Yongyong Feng^1, 2^, Zhenlu Wang^1, 2^ , Wenzhao Jiang^1, 2^ , Yuexin Qu^1, 2^, Chaonan Zhang^1, 2^, Aiguo Zhou^1, 2^, Shaolin Xie^1, 2^, and Jixing Zou^1, 2^

^1^ Healthy Aquaculture Laboratory, College of Marine Sciences, South China Agricultural University, Guangzhou, Guangdong, China.

^2^ Joint Laboratory of Guangdong Province and Hong Kong Region on Marine Bioresource Conservation and Exploitation, College of Marine Sciences, South China Agricultural University, Guangzhou, Guangdong, China.

Corresponding author: J. X. Zou. E-mail: zoujixing@scau.edu.cn.

**Supplementary table S1** Primers sequences used in RT-qPCR.

| Primer Name | Sequence (5' to 3') | Tm (℃) | Purpose |
| --- | --- | --- | --- |
| MTT2/4 | F: ATCCCTGCTCTTGTAATCCC | 54.1 | RT-qPCR |
|  | R: AGTTGGAAGTAGAACCGCA |  |  |
| HSP70 | F:TGAGAATCATCAACGAACCCAC | 54.9 | RT-qPCR |
|  | R:CGAAGATACCGTCATCAAGAGTAA |  |  |
| CYP1 | F: AGTGATTATTGCCTCATTCTTTGG | 51.2 | RT-qPCR |
|  | R: TGTTCTTCAGTAACCCCTAATTCG |  |  |
| MPK1 | F:ATCCGAAAGCAAATCCACT | 52.1 | RT-qPCR |
|  | R:TCAGGTTCTTCATCAGGGT |  |  |
| MPK3 | F:AAGGGTTTGAAGTATCTCC | 51.2 | RT-qPCR |
|  | R:CAGCCTTTGAGTAAACATG |  |  |
| ATG7 | F:CAGTTACTCGTCCTGGTT | 51 | RT-qPCR |
|  | R:CTCTGCCATACATCACTCT |  |  |
| 18S rRNA | F: CCTGGGAAGGTACGGGTAAT | 53.7 | RT-qPCR |
|  | R: AAGGTTCACCAGACCATTCG |  |  |

**Supplementary table S2** Cells densities of different times and concentrations of gelsemine in *Tetrahymena thermophila*

| processing time | Blank control | Negative control | 0.05 mg/mL | 0.1 mg/mL | 0.2 mg/mL | 0.4 mg/mL | 0.8 mg/mL |
| --- | --- | --- | --- | --- | --- | --- | --- |
|  | Least-squares mean±SEM (×10^5^ cells/mL) | | | | | | |
| 12h | 1.41±0.04a | 1.42±.031a | 1.28±0.06a | 1.22±0.03b | 1.14±0.12b | 0.94±0.09c | 0.97±0.08c |
| 24h | 3.03±0.17a | 2.75±0.06a | 2.53±0.15b | 1.98±0.08c | 1.78±0.9c | 1.73±0.10c | 1.66±0.08d |
| 36h | 3.59±0.20a | 3.54±0.20a | 3.54±0.18a | 2.35±0.12b | 2.09±0.09b | 2.06±0.10b | 1.82±0.08c |
| 48h | 5.21±0.21a | 4.78±0.14a | 4.29±0.08b | 3.63±0.16c | 2.64±0.14d | 2.50±0.10d | 2.32±0.21d |
| 60h | 5.53±0.21a | 4.91±0.04a | 3.94±0.14b | 3.40±0.13c | 2.71±0.08d | 2.44±0.08d | 2.22±0.09e |
| 72h | 5.60±0.22a | 5.10±0.10b | 3.56±0.14c | 3.51±0.15c | 3.35±0.12c | 2.33±0.15d | 2.26±0.14d |

**Notes.**

Data are summarized as means ±SEM

Values within a row with no common superscript differ significantly (*P* < 0.05) or are highly significant (*P* < 0.01).

**Supplementary table S3** Cells in different DNA damage classes measured in *T. thermophila* exposed to Gelsemine (calculated on the basis of DNA percentage in the comet tail).

| Damage classes | Blank control | NC | Gelsemine (mg/mL) | | | | |
| --- | --- | --- | --- | --- | --- | --- | --- |
|  |  |  | 0.05 | 0.1 | 0.2 | 0.4 | 0.8 |
| Min | 110 | 113 | 91 | 89 | 57 | 18 | 3 |
| Low | 29 | 26 | 38 | 49 | 64 | 33 | 20 |
| Mid | 4 | 3 | 16 | 13 | 31 | 66 | 71 |
| High | 1 | 0 | 1 | 1 | 1 | 21 | 44 |
| Extreme | 0 | 0 | 0 | 0 | 0 | 0 | 4 |

**Notes.**

Min = minimal damage <20% DNA in the comet tail; Low = low damage 20-40% DNA in the comet tail; Mid = mid damage 40-60% DNA in the comet tail; High = high damage 60-80% DNA in the comet tail and Extreme = extreme damage >80% DNA in the comet tail.

**Supplementary table S3** Raw data of DNA damage exposed to a various levels of Gelsemine.

| Blank control | | Negative control | | 0.05 mg/mL | | 0.1 mg/mL | | 0.2 mg/mL | | 0.4 mg/mL | | 0.8 mg/mL | |
| --- | --- | --- | --- | --- | --- | --- | --- | --- | --- | --- | --- | --- | --- |
| TailDNA% | OTM | TailDNA% | OTM | TailDNA% | OTM | TailDNA% | OTM | TailDNA% | OTM | TailDNA% | OTM | TailDNA% | OTM |
| 17.3467 | 14.6276 | 19.379 | 23.0387 | 17.0362 | 13.3351 | 17.6675 | 21.0433 | 17.6675 | 21.0433 | 18.7334 | 25.5683 | 51.1275 | 32.5259 |
| 17.4743 | 16.0626 | 19.2883 | 15.8202 | 18.1493 | 14.2302 | 18.1493 | 14.2302 | 18.1708 | 19.7389 | 41.298 | 26.6943 | 58.8668 | 40.6622 |
| 17.6073 | 16.7188 | 19.2948 | 8.16339 | 19.9511 | 16.1157 | 19.3322 | 15.8203 | 18.6447 | 20.6586 | 43.1289 | 28.6679 | 63.0239 | 44.3858 |
| 18.0692 | 16.9187 | 19.826 | 7.32762 | 15.076 | 12.4885 | 19.6447 | 20.6586 | 19.1289 | 28.6679 | 47.4594 | 27.4529 | 63.8857 | 35.8652 |
| 18.1424 | 17.1875 | 19.9296 | 12.7747 | 16.9897 | 11.6042 | 45.2207 | 20.2195 | 19.4594 | 27.4529 | 48.5228 | 27.5880 | 67.8526 | 34.4015 |
| 18.265 | 14.9211 | 13.9764 | 10.0921 | 17.3199 | 12.4444 | 19.6953 | 14.5133 | 38.5228 | 27.5880 | 51.1275 | 32.5259 | 20.1073 | 33.6414 |
| 18.4207 | 12.3872 | 18.2184 | 10.9164 | 19.6953 | 14.5133 | 23.9511 | 16.1157 | 26.0929 | 20.1252 | 58.8668 | 40.6622 | 27.6248 | 31.7881 |
| 18.5108 | 12.7254 | 29.8319 | 13.5929 | 21.3322 | 15.8203 | 26.0929 | 20.1252 | 26.2579 | 21.5119 | 63.0239 | 44.3858 | 29.8016 | 32.4128 |
| 18.5933 | 17.3879 | 36.9595 | 17.3334 | 29.3375 | 18.6590 | 26.2579 | 21.5119 | 29.3375 | 18.6590 | 63.8857 | 35.8652 | 36.0507 | 37.1277 |
| 18.7525 | 16.101 | 42.6588 | 16.078 | 34.1171 | 19.2123 | 29.3375 | 18.6590 | 34.1171 | 19.2123 | 67.8526 | 34.4015 | 41.3984 | 31.3266 |
| 18.7551 | 16.2819 | 18.018 | 18.2412 | 41.1708 | 19.7389 | 34.1171 | 19.2123 | 37.2207 | 20.2195 | 18.1378 | 23.6926 | 55.6524 | 32.5802 |
| 18.7746 | 17.8267 | 18.6185 | 11.9995 | 18.1122 | 14.9456 | 41.1708 | 19.7389 | 38.7334 | 25.5683 | 18.3009 | 29.3565 | 58.9646 | 45.7672 |
| 18.826 | 7.32762 | 18.9739 | 18.8929 | 18.2206 | 13.8914 | 46.1745 | 20.9989 | 41.298 | 26.6943 | 18.5125 | 30.7454 | 59.9011 | 40.6833 |
| 18.9059 | 11.7139 | 19.0869 | 16.501 | 18.3273 | 19.9107 | 18.1122 | 14.9456 | 41.8792 | 24.8619 | 18.8858 | 21.3049 | 60.6761 | 43.8696 |
| 18.9122 | 18.1623 | 19.2713 | 15.0044 | 18.6284 | 19.0698 | 18.1368 | 17.9974 | 46.1745 | 20.9989 | 32.9059 | 28.4294 | 62.626 | 50.8556 |
| 18.9839 | 14.8632 | 19.3828 | 11.2155 | 18.8397 | 16.9957 | 18.2206 | 13.8914 | 17.6662 | 19.2275 | 35.3284 | 29.3849 | 63.4399 | 40.9462 |
| 19.0756 | 17.0775 | 21.5035 | 18.7077 | 18.8805 | 16.5236 | 18.4100 | 15.7933 | 17.8858 | 21.3049 | 37.0709 | 29.8281 | 64.6411 | 39.5664 |
| 19.0805 | 15.6411 | 17.5339 | 12.0872 | 19.6662 | 19.2275 | 18.8805 | 16.5236 | 18.1368 | 17.9974 | 38.861 | 31.0590 | 66.8639 | 49.4411 |
| 19.1809 | 7.16253 | 10.6905 | 6.55896 | 16.7549 | 11.9107 | 19.0674 | 16.9361 | 18.3273 | 19.9107 | 40.1073 | 33.6414 | 81.6118 | 51.7823 |
| 19.2473 | 16.3885 | 14.1346 | 6.5613 | 17.2036 | 12.6737 | 19.2261 | 20.0226 | 18.3284 | 29.3849 | 41.0687 | 30.5869 | 22.6995 | 32.4313 |
| 19.2901 | 16.1626 | 15.4455 | 9.01332 | 20.5924 | 15.3144 | 19.8397 | 16.9957 | 19.0709 | 29.8281 | 41.3984 | 31.3266 | 25.8781 | 32.9274 |
| 19.2948 | 8.16339 | 19.296 | 16.7277 | 22.41 | 15.7933 | 20.5924 | 15.3144 | 19.3984 | 31.3266 | 43.3753 | 25.4294 | 37.532 | 34.2438 |
| 19.2953 | 18.8605 | 19.672 | 15.8865 | 24.1368 | 17.9974 | 25.6284 | 19.0698 | 19.9059 | 28.4294 | 46.0507 | 37.1277 | 50.4128 | 35.6976 |
| 19.298 | 11.7088 | 3.06885 | 2.50297 | 26.0674 | 16.9361 | 26.8858 | 21.3049 | 33.3753 | 25.4294 | 47.6248 | 31.7881 | 50.5243 | 38.0034 |
| 19.3501 | 14.7255 | 30.1927 | 17.8409 | 26.9276 | 13.2653 | 27.6662 | 19.2275 | 37.6248 | 31.7881 | 49.8016 | 32.4128 | 54.2846 | 35.6155 |
| 19.3936 | 14.1566 | 19.1388 | 18.8319 | 18.8179 | 16.7806 | 35.8926 | 20.4273 | 25.6284 | 19.0698 | 55.6524 | 32.5802 | 54.6668 | 39.4552 |
| 19.5153 | 16.9621 | 4.41493 | 3.14028 | 19.1142 | 18.4614 | 36.3273 | 19.9107 | 29.2261 | 20.0226 | 58.9646 | 45.7672 | 55.1894 | 32.3857 |
| 19.5332 | 10.0379 | 4.72673 | 3.95051 | 19.3508 | 15.2244 | 17.3508 | 15.2244 | 35.1378 | 23.6926 | 59.9011 | 40.6833 | 62.3726 | 40.6563 |
| 19.6258 | 4.72782 | 55.5757 | 16.2482 | 19.5756 | 15.7379 | 17.5489 | 21.9692 | 35.3009 | 29.3565 | 60.6761 | 43.8696 | 65.9953 | 39.8382 |
| 19.6333 | 17.0628 | 19.0764 | 17.4898 | 19.7727 | 17.4262 | 18.6635 | 19.4665 | 35.8926 | 20.4273 | 63.4399 | 40.9462 | 66.7347 | 43.5177 |
| 19.656 | 17.2358 | 19.1795 | 15.6728 | 12.8435 | 11.6397 | 18.7727 | 17.4262 | 36.5125 | 30.7454 | 64.6411 | 39.5664 | 69.4476 | 48.1395 |
| 19.6945 | 11.1477 | 19.9525 | 11.8322 | 17.6526 | 13.8319 | 19.1142 | 18.4614 | 38.861 | 31.0590 | 19.1207 | 21.6735 | 70.4375 | 43.3990 |
| 19.72 | 18.1912 | 11.9797 | 9.1462 | 17.7422 | 12.6594 | 19.1207 | 21.6735 | 41.0687 | 30.5869 | 25.3981 | 22.2091 | 70.4627 | 43.4349 |
| 19.7763 | 15.7518 | 14.1367 | 13.7609 | 17.9138 | 14.5942 | 19.3981 | 22.2091 | 18.1142 | 18.4614 | 27.5489 | 21.9692 | 24.0162 | 33.0071 |
| 19.795 | 17.3943 | 14.6944 | 7.68388 | 25.2207 | 14.4048 | 19.4609 | 21.9880 | 18.6843 | 22.3954 | 30.6843 | 22.3954 | 27.7707 | 36.2723 |
| 19.807 | 13.1779 | 16.2935 | 12.9714 | 27.9258 | 18.9490 | 19.6843 | 22.3954 | 18.9258 | 18.9490 | 32.4609 | 21.9880 | 84.0047 | 55.9404 |
| 19.8154 | 18.4809 | 17.9857 | 12.6234 | 31.6635 | 19.4665 | 19.8179 | 16.7806 | 19.4860 | 25.9373 | 37.532 | 34.2438 | 48.7902 | 35.4422 |
| 19.8369 | 15.2755 | 33.9045 | 16.5938 | 15.3493 | 18.2971 | 17.6526 | 13.8319 | 33.4948 | 28.9442 | 38.486 | 25.9373 | 48.8517 | 32.0567 |
| 19.8667 | 16.2285 | 58.8442 | 15.8694 | 18.2972 | 17.8909 | 17.9138 | 14.5942 | 25.3981 | 22.2091 | 39.2022 | 23.3205 | 49.9934 | 35.9941 |
| 19.8985 | 9.79923 | 7.07339 | 5.70198 | 19.9788 | 18.5633 | 25.2207 | 14.4048 | 27.5489 | 21.9692 | 39.9589 | 26.0217 | 51.0061 | 36.9032 |
| 19.9264 | 10.4465 | 19.9323 | 16.8015 | 15.5907 | 12.2771 | 27.5756 | 15.7379 | 30.1207 | 21.6735 | 40.1834 | 28.2007 | 53.4816 | 38.6109 |
| 19.9296 | 12.7747 | 28.8985 | 17.9105 | 17.0417 | 13.8528 | 27.9258 | 18.9490 | 31.6635 | 19.4665 | 41.4121 | 26.0244 | 54.2563 | 47.9039 |
| 19.9552 | 16.2484 | 18.2038 | 13.5078 | 23.2749 | 16.6247 | 39.2022 | 23.3205 | 32.4609 | 21.9880 | 42.6995 | 32.4313 | 59.8778 | 36.9420 |
| 19.9767 | 15.5688 | 35.5676 | 23.0833 | 24.5093 | 19.2220 | 17.9788 | 18.5633 | 39.2022 | 23.3205 | 43.4948 | 28.9442 | 64.7945 | 34.6347 |
| 21.0505 | 18.4089 | 18.1801 | 16.9522 | 25.7265 | 18.9856 | 18.2749 | 16.6247 | 39.9589 | 26.0217 | 45.4565 | 26.4760 | 18.2106 | 31.4889 |
| 21.8446 | 18.4211 | 19.2934 | 12.3316 | 30.8931 | 18.7221 | 18.2972 | 17.8909 | 40.1834 | 28.2007 | 45.8781 | 32.9274 | 82.3698 | 55.6924 |
| 22.0055 | 16.6985 | 19.425 | 16.7411 | 31.2397 | 17.4385 | 18.4728 | 16.4045 | 41.4121 | 26.0244 | 50.4128 | 35.6976 | 44.3349 | 43.3777 |
| 22.9316 | 17.9819 | 19.654 | 16.9069 | 31.4728 | 16.4045 | 18.5131 | 22.4580 | 45.4565 | 26.4760 | 50.5243 | 38.0034 | 51.5109 | 37.4554 |
| 23.0933 | 18.3279 | 19.9824 | 17.1196 | 31.6706 | 19.8450 | 18.8944 | 22.6306 | 18.2972 | 17.8909 | 54.2846 | 35.6155 | 52.3969 | 48.8226 |
| 23.1632 | 18.3103 | 13.0743 | 9.40336 | 37.762 | 19.1381 | 19.3493 | 18.2971 | 18.3493 | 18.2971 | 54.6668 | 39.4552 | 52.9021 | 38.8814 |
| 24.295 | 18.0058 | 13.1694 | 9.4699 | 37.9144 | 19.1179 | 19.7265 | 18.9856 | 18.4070 | 22.9466 | 55.1894 | 32.3857 | 55.174 | 33.7180 |
| 24.7497 | 12.0672 | 13.2926 | 10.1045 | 18.5745 | 13.8201 | 19.7620 | 19.1381 | 18.5093 | 19.2220 | 62.3726 | 40.6563 | 61.9662 | 46.9201 |
| 25.3269 | 16.0911 | 14.6007 | 11.3246 | 18.9296 | 12.7747 | 24.5093 | 19.2220 | 18.9788 | 18.5633 | 65.9953 | 39.8382 | 63.4687 | 34.5974 |
| 25.7582 | 11.316 | 14.7254 | 9.43097 | 18.9624 | 17.5804 | 41.2397 | 17.4385 | 19.0286 | 30.7569 | 66.7347 | 43.5177 | 67.3559 | 55.4036 |
| 10.4476 | 6.94114 | 18.2935 | 13.2361 | 19.1974 | 18.0049 | 17.0417 | 13.8528 | 19.0571 | 25.5617 | 70.4375 | 43.3990 | 71.4633 | 33.2433 |
| 11.3652 | 7.36823 | 19.0176 | 14.6199 | 19.3576 | 16.8829 | 30.5814 | 22.3393 | 19.3790 | 23.0387 | 70.4627 | 43.4349 | 37.5017 | 31.8019 |
| 11.6013 | 8.16236 | 19.4215 | 14.5531 | 19.4712 | 18.2065 | 30.8931 | 18.7221 | 19.6706 | 19.8450 | 17.3493 | 18.2971 | 38.5029 | 33.2073 |
| 11.6506 | 8.08307 | 29.649 | 17.9095 | 19.5353 | 17.8916 | 31.6706 | 19.8450 | 19.6718 | 26.3490 | 18.1760 | 24.0790 | 42.6895 | 31.7718 |
| 11.7342 | 8.04115 | 33.6883 | 15.3386 | 19.6591 | 18.1372 | 32.8478 | 22.2183 | 19.7265 | 18.9856 | 19.3790 | 23.0387 | 50.1381 | 34.9491 |
| 11.7633 | 8.21009 | 18.5909 | 16.5651 | 13.4703 | 11.8124 | 37.379 | 23.0387 | 19.9144 | 19.1179 | 19.5131 | 22.4580 | 50.6955 | 34.0721 |
| 12.0333 | 8.15925 | 19.1981 | 17.6447 | 17.0698 | 12.6905 | 37.9144 | 19.1179 | 28.176 | 24.0790 | 19.6718 | 26.3490 | 52.3511 | 32.9914 |
| 12.0714 | 8.77229 | 19.2732 | 11.0577 | 17.2838 | 14.9973 | 39.407 | 22.9466 | 29.8944 | 22.6306 | 19.8478 | 22.2183 | 53.5483 | 44.8930 |
| 12.9739 | 5.61181 | 19.935 | 16.3259 | 17.7796 | 14.9216 | 42.1255 | 21.0752 | 30.5814 | 22.3393 | 19.8944 | 22.6306 | 54.5956 | 32.1448 |
| 13.2114 | 9.51025 | 25.2911 | 16.982 | 19.8362 | 15.2979 | 17.5353 | 17.8916 | 30.8931 | 18.7221 | 30.0571 | 25.5617 | 62.0166 | 48.7630 |
| 13.2528 | 8.30483 | 15.2104 | 11.4336 | 28.5133 | 15.3355 | 18.6591 | 18.1372 | 32.8478 | 22.2183 | 30.5814 | 22.3393 | 64.8703 | 56.7726 |
| 13.8851 | 9.75001 | 16.3387 | 11.3847 | 16.0542 | 17.4376 | 18.9624 | 17.5804 | 33.5131 | 22.4580 | 36.9913 | 28.2736 | 68.8413 | 39.9822 |
| 13.9764 | 10.0921 | 17.9414 | 11.5865 | 19.5339 | 12.0872 | 19.1974 | 18.0049 | 36.9913 | 28.2736 | 37.7707 | 36.2723 | 70.577 | 38.4913 |
| 14.1937 | 4.01236 | 19.4311 | 15.7258 | 19.5696 | 19.8338 | 19.3576 | 16.8829 | 37.762 | 19.1381 | 39.0286 | 30.7569 | 26.1326 | 31.4146 |
| 14.5201 | 13.6205 | 19.9058 | 11.2948 | 24.0954 | 16.6155 | 19.4712 | 18.2065 | 39.1149 | 28.2475 | 39.1149 | 28.2475 | 28.6632 | 34.2988 |
| 14.598 | 9.73456 | 29.1017 | 17.7258 | 17.0075 | 13.5355 | 17.2838 | 14.9973 | 39.3687 | 25.2868 | 39.3687 | 25.2868 | 46.3561 | 39.7834 |
| 15.3786 | 9.61681 | 19.5333 | 17.8535 | 19.6341 | 12.8493 | 17.7796 | 14.9216 | 42.1255 | 21.0752 | 39.407 | 22.9466 | 51.072 | 43.4123 |
| 15.8155 | 13.8545 | 19.7424 | 17.8992 | 17.5369 | 13.7471 | 19.8362 | 15.2979 | 45.4635 | 31.2497 | 44.0162 | 33.0071 | 59.1765 | 39.4467 |
| 15.8514 | 6.91077 | 5.48642 | 4.05374 | 18.4968 | 12.9339 | 22.5745 | 13.8201 | 48.8517 | 32.0567 | 45.4635 | 31.2497 | 61.5641 | 40.7602 |
| 16.3278 | 10.7156 | 5.92437 | 4.61829 | 18.5369 | 13.7471 | 28.5133 | 15.3355 | 52.82 | 29.8625 | 48.7902 | 35.4422 | 65.1474 | 42.3447 |
| 16.4217 | 8.94215 | 18.0674 | 14.9409 | 18.6461 | 17.3088 | 29.3293 | 22.3844 | 18.0050 | 21.8683 | 48.8517 | 32.0567 | 71.9738 | 47.5315 |
| 16.556 | 13.6289 | 19.2603 | 8.78819 | 18.9876 | 19.9886 | 31.005 | 21.8683 | 19.3293 | 22.3844 | 49.9934 | 35.9941 | 77.3338 | 47.3703 |
| 16.8716 | 9.28336 | 21.3378 | 18.0232 | 15.8279 | 12.1672 | 36.858 | 22.6126 | 19.3576 | 29.3679 | 51.0061 | 36.9032 | 53.2953 | 34.4025 |
| 16.89 | 12.9293 | 22.8084 | 18.4244 | 15.8279 | 12.1672 | 18.0954 | 16.6155 | 19.4712 | 18.2065 | 52.82 | 29.8625 | 58.8925 | 50.5562 |
| 16.9249 | 13.62 | 19.3185 | 15.4412 | 19.6358 | 17.0252 | 18.1819 | 20.1409 | 19.6591 | 18.1372 | 53.4816 | 38.6109 | 61.7413 | 38.2812 |
| 16.9289 | 11.0658 | 28.0484 | 15.8164 | 22.9525 | 11.8322 | 18.5696 | 19.8338 | 23.5353 | 17.8916 | 54.2563 | 47.9039 | 63.1508 | 41.9930 |
| 16.9897 | 11.6042 | 10.193 | 7.82375 | 58.8442 | 15.8694 | 19.0542 | 17.4376 | 24.1974 | 18.0049 | 59.8778 | 36.9420 | 65.3387 | 41.3234 |
| 17.2337 | 13.1509 | 15.9073 | 13.4427 | 18.7061 | 17.0724 | 17.0075 | 13.5355 | 36.858 | 22.6126 | 64.7945 | 34.6347 | 52.5809 | 32.9435 |
| 17.3403 | 9.98844 | 19.8623 | 13.7719 | 19.4867 | 16.3503 | 17.5369 | 13.7471 | 38.2106 | 31.4889 | 18.2106 | 31.4889 | 55.5025 | 37.1541 |
| 17.4325 | 12.6426 | 18.5687 | 15.3506 | 18.2038 | 13.5078 | 17.5369 | 13.7471 | 55.7877 | 27.7933 | 19.0050 | 21.8683 | 57.2899 | 35.5627 |
| 17.483 | 10.8794 | 18.5906 | 15.3807 | 19.9489 | 14.8488 | 18.4138 | 21.9386 | 19.1819 | 20.1409 | 29.3293 | 22.3844 | 57.6579 | 39.9766 |
| 17.4986 | 13.4554 | 18.6232 | 14.1017 | 20.5053 | 16.3460 | 18.6461 | 17.3088 | 19.2674 | 24.7165 | 36.858 | 22.6126 | 62.8749 | 42.3679 |
| 17.6153 | 8.47693 | 18.9744 | 10.849 | 20.7763 | 17.7509 | 18.9876 | 19.9886 | 19.3775 | 24.9227 | 39.3576 | 29.3679 | 63.8805 | 53.8895 |
| 17.6587 | 12.782 | 19.0293 | 14.9285 | 23.0297 | 17.1290 | 19.2568 | 23.1521 | 19.7188 | 25.5998 | 44.3349 | 43.3777 | 71.1958 | 39.5938 |
| 17.6976 | 14.6142 | 19.2184 | 17.8405 | 30.5 | 19.5453 | 68.8442 | 15.8694 | 19.7189 | 24.5385 | 51.5109 | 37.4554 | 19.4398 | 32.1339 |
| 17.8053 | 10.3071 | 19.3594 | 9.51237 | 18.8910 | 18.0646 | 19.6358 | 17.0252 | 19.8599 | 25.0744 | 52.9021 | 38.8814 | 25.3152 | 32.9104 |
| 17.9293 | 11.2863 | 19.9104 | 15.411 | 15.0458 | 12.1933 | 18.4867 | 16.3503 | 36.0270 | 27.6441 | 55.174 | 33.7180 | 86.8872 | 61.5279 |
| 18.2113 | 9.5613 | 21.2123 | 18.3517 | 21.189 | 15.0431 | 18.5053 | 16.3460 | 37.3481 | 27.6061 | 55.7877 | 27.7933 | 50.0917 | 35.7125 |
| 18.2184 | 10.9164 | 24.7048 | 15.8911 | 23.3476 | 16.6045 | 18.5996 | 21.2531 | 29.5696 | 19.8338 | 61.9662 | 46.9201 | 57.1215 | 49.2249 |
| 18.2809 | 12.3742 | 15.1542 | 8.68163 | 30.2616 | 18.6342 | 18.7061 | 17.0724 | 41.3107 | 26.0530 | 63.4687 | 34.5974 | 60.332 | 47.5443 |
| 18.5906 | 14.3895 | 15.9974 | 10.4821 | 32.526 | 16.1639 | 19.4153 | 20.8706 | 42.5944 | 28.2403 | 71.4633 | 33.2433 | 63.0276 | 36.4010 |
| 19.1403 | 15.4324 | 16.106 | 12.4188 | 18.5542 | 17.3973 | 44.4761 | 21.2333 | 42.6895 | 31.7718 | 19.3775 | 24.9227 | 35.3720 | 34.3590 |
| 19.1667 | 15.2468 | 16.6372 | 14.1702 | 18.7715 | 15.2903 | 18.2038 | 13.5078 | 42.8084 | 25.9382 | 33.7189 | 24.5385 | 47.9793 | 31.6275 |
| 19.2912 | 10.7174 | 18.6757 | 11.6878 | 19.7864 | 17.7909 | 19.9489 | 14.8488 | 46.1823 | 29.5431 | 35.2674 | 24.7165 | 53.7051 | 31.8304 |
| 19.6051 | 13.4069 | 4.84251 | 2.4917 | 26.0372 | 17.7537 | 20.7763 | 17.7509 | 47.2976 | 29.2956 | 36.7188 | 25.5998 | 57.676 | 37.7052 |
| 19.7899 | 15.5947 | 5.33514 | 3.27484 | 26.8335 | 16.8076 | 23.0297 | 17.1290 | 47.5017 | 31.8019 | 38.5029 | 33.2073 | 59.8281 | 42.8428 |
| 19.8444 | 16.41 | 9.9022 | 7.09042 | 17.6860 | 19.3139 | 27.4153 | 21.2284 | 48.1782 | 30.9728 | 38.8599 | 25.0744 | 60.421 | 47.7360 |
| 19.8866 | 12.4187 | 17.9623 | 13.7282 | 18.0153 | 18.4965 | 30.5 | 19.5453 | 52.467 | 27.8668 | 41.3107 | 26.0530 | 66.7109 | 36.1819 |
| 19.891 | 15.7914 | 29.631 | 13.7442 | 14.6073 | 13.1522 | 40.8464 | 21.7041 | 54.5956 | 32.1448 | 42.5944 | 28.2403 | 45.6661 | 36.1743 |
| 2.88458 | 2.2366 | 19.3698 | 14.3922 | 16.4347 | 11.6318 | 18.8910 | 18.0646 | 18.9495 | 28.3590 | 42.6895 | 31.7718 | 51.1765 | 32.9208 |
| 19.177 | 16.3368 | 2.93343 | 2.32698 | 19.9347 | 12.3676 | 19.0337 | 21.1797 | 19.3454 | 26.7152 | 42.8084 | 25.9382 | 52.3308 | 40.6867 |
| 19.3394 | 11.7478 | 18.0106 | 15.9317 | 24.5159 | 16.9410 | 19.2820 | 20.1329 | 19.4138 | 21.9386 | 46.027 | 27.6441 | 53.0222 | 32.2416 |
| 19.4547 | 15.3039 | 18.0371 | 12.3342 | 27.6579 | 17.7330 | 21.189 | 15.0431 | 19.5084 | 29.1248 | 46.1823 | 29.5431 | 53.0981 | 35.5537 |
| 19.5797 | 6.51893 | 18.3124 | 18.6735 | 38.0484 | 15.8164 | 23.3476 | 16.6045 | 22.9876 | 19.9886 | 47.2976 | 29.2956 | 53.2928 | 38.9759 |
| 19.6933 | 10.1343 | 19.3303 | 11.9304 | 40.2154 | 16.5663 | 30.2616 | 18.6342 | 33.2568 | 23.1521 | 47.3481 | 27.6061 | 53.9999 | 37.1920 |
| 19.9118 | 14.1221 | 19.6157 | 13.3037 | 17.8663 | 19.0824 | 32.526 | 16.1639 | 36.1326 | 31.4146 | 47.5017 | 31.8019 | 54.1934 | 36.8302 |
| 21.6107 | 16.6856 | 19.6327 | 17.2541 | 18.3130 | 12.9889 | 18.4669 | 21.5576 | 43.2121 | 30.2226 | 48.1782 | 30.9728 | 56.183 | 34.2368 |
| 21.7271 | 13.0453 | 19.6776 | 15.4481 | 18.3270 | 12.8706 | 18.7715 | 15.2903 | 43.4919 | 30.3392 | 50.1381 | 34.9491 | 66.2273 | 45.6664 |
| 21.8325 | 16.8035 | 19.9372 | 16.4361 | 18.6893 | 17.7043 | 19.0372 | 17.7537 | 47.715 | 28.0686 | 50.6955 | 34.0721 | 71.6601 | 37.7922 |
| 29.8952 | 16.8406 | 19.9906 | 10.1093 | 18.8951 | 18.6899 | 41.5542 | 17.3973 | 47.7833 | 27.1190 | 52.3511 | 32.9914 | 21.4511 | 33.9029 |
| 3.74524 | 2.64715 | 22.2837 | 15.4996 | 18.9042 | 16.8272 | 25.7864 | 17.7909 | 18.4761 | 21.2333 | 52.467 | 27.8668 | 31.8385 | 32.9120 |
| 25.0616 | 16.7945 | 23.1996 | 12.3672 | 19.0206 | 18.9245 | 26.8335 | 16.8076 | 18.5000 | 19.5453 | 53.5483 | 44.8930 | 52.5377 | 35.3408 |
| 30.6014 | 21.2054 | 26.3709 | 17.9821 | 16.7503 | 11.7140 | 29.1674 | 22.7694 | 18.5996 | 21.2531 | 54.5956 | 32.1448 | 52.8991 | 31.4795 |
| 30.7817 | 21.2114 | 14.7797 | 11.8288 | 17.3382 | 11.7830 | 17.6579 | 17.7330 | 19.4153 | 20.8706 | 68.8413 | 39.9822 | 56.737 | 42.6436 |
| 30.9343 | 9.98494 | 15.2977 | 9.33434 | 18.1665 | 12.2472 | 18.0484 | 15.8164 | 33.3374 | 30.7386 | 70.577 | 38.4913 | 59.7439 | 50.6554 |
| 31.349 | 17.0096 | 17.8482 | 12.0974 | 19.4484 | 13.3669 | 18.5159 | 16.9410 | 40.8464 | 21.7041 | 18.9495 | 28.3590 | 43.2341 | 34.6613 |
| 32.127 | 21.1309 | 18.8082 | 12.6069 | 21.0378 | 13.2301 | 19.6860 | 19.3139 | 20.7763 | 17.7509 | 19.2568 | 23.1521 | 47.4909 | 32.7278 |
| 32.3911 | 18.377 | 29.6114 | 17.3372 | 23.8005 | 15.6547 | 19.7496 | 21.0534 | 27.4153 | 21.2284 | 31.4138 | 21.9386 | 54.9029 | 35.8131 |
| 32.7759 | 18.1469 | 30.9505 | 16.727 | 25.4361 | 16.2183 | 19.9602 | 22.3463 | 31.7092 | 23.9627 | 33.5084 | 29.1248 | 57.866 | 49.3943 |
| 33.3392 | 17.402 | 19.9103 | 16.2098 | 27.2728 | 18.7108 | 24.0153 | 18.4965 | 35.9804 | 28.4273 | 36.1326 | 31.4146 | 58.5011 | 44.8163 |
| 24.0511 | 10.6633 | 17.0097 | 14.791 | 37.7807 | 19.1965 | 31.2618 | 22.1074 | 17.2616 | 18.6342 | 38.6632 | 34.2988 | 65.9386 | 31.9921 |
| 24.3081 | 9.21685 | 17.8293 | 14.5055 | 18.1306 | 14.7134 | 40.2154 | 16.5663 | 18.8970 | 25.4511 | 40.3454 | 26.7152 | 66.9094 | 47.5496 |
| 28.0957 | 18.3497 | 19.5837 | 14.4487 | 19.2440 | 14.0374 | 17.8005 | 15.6547 | 19.0327 | 24.4873 | 43.2121 | 30.2226 | 26.7390 | 34.3223 |
| 28.679 | 22.6615 | 28.8139 | 10.8763 | 18.7636 | 13.6385 | 18.6130 | 22.2814 | 19.0337 | 21.1797 | 43.4919 | 30.3392 | 39.2255 | 32.4003 |
| 39.4176 | 11.2415 | 11.272 | 9.87447 | 24.7367 | 14.9567 | 18.8951 | 18.6899 | 28.891 | 18.0646 | 46.3561 | 39.7834 | 44.5644 | 33.1494 |
| 29.5013 | 16.0573 | 17.0811 | 2.97041 | 29.362 | 16.1541 | 19.0206 | 18.9245 | 30.282 | 20.1329 | 47.715 | 28.0686 | 47.814 | 33.8647 |
| 4.48872 | 3.3979 | 18.6619 | 10.6522 | 41.3832 | 13.4537 | 19.2728 | 18.7108 | 43.6549 | 30.1675 | 47.7833 | 27.1190 | 49.8865 | 38.1902 |
| 4.63588 | 3.25958 | 18.7551 | 9.8511 | 17.9888 | 11.8720 | 19.4361 | 16.2183 | 65.9857 | 29.3761 | 51.072 | 43.4123 | 55.5302 | 43.9695 |
| 41.1057 | 14.874 | 19.2814 | 9.45237 | 19.9376 | 12.2477 | 19.8663 | 19.0824 | 18.5058 | 24.6983 | 59.1765 | 39.4467 | 59.407 | 44.4990 |
| 42.2693 | 22.6601 | 30.2763 | 13.8946 | 36.6334 | 11.6563 | 40.7871 | 21.9704 | 18.7313 | 24.8313 | 61.5641 | 40.7602 | 68.5457 | 52.5608 |
| 45.6493 | 14.2654 | 34.1262 | 18.8293 | 17.8401 | 16.1731 | 47.0499 | 23.2643 | 19.0372 | 17.7537 | 65.1474 | 42.3447 | 69.6647 | 36.1998 |
| 47.5127 | 18.6831 | 19.6252 | 15.4813 | 39.9315 | 18.5469 | 47.7807 | 19.1965 | 19.1674 | 22.7694 | 71.9738 | 47.5315 | 46.9068 | 40.3470 |
| 5.64408 | 3.45827 | 11.5547 | 4.34657 | 18.7995 | 19.3328 | 21.6893 | 17.7043 | 19.4669 | 21.5576 | 77.3338 | 47.3703 | 48.345 | 38.0371 |
| 5.73325 | 5.34429 | 11.8163 | 4.58127 | 17.2801 | 16.0748 | 21.9042 | 16.8272 | 25.7864 | 17.7909 | 23.1133 | 18.2889 | 18.3388 | 31.4073 |
| 6.29028 | 3.85025 | 14.4595 | 12.1979 | 18.1996 | 12.3672 | 29.6771 | 21.4490 | 35.4398 | 32.1339 |  |  | 57.9632 | 42.7802 |
| 6.53511 | 4.97642 | 9.5412 | 7.09745 | 18.2567 | 16.6728 | 42.3829 | 21.8106 | 40.1683 | 31.1912 |  |  | 28.1435 | 37.3451 |
| 6.72244 | 5.08155 | 43.2678 | 23.0439 | 19.4776 | 15.8057 | 18.2440 | 14.0374 | 42.6571 | 30.6837 |  |  | 65.2527 | 48.3279 |
| 63.9694 | 13.3077 | 19.2015 | 23.0984 | 19.7466 | 15.8458 | 19.3620 | 16.1541 | 34.0035 | 27.7333 |  |  | 56.0332 | 34.5828 |
| 7.97466 | 3.41906 | 34.1637 | 23.1228 | 16.5843 | 12.5143 | 18.7636 | 13.6385 | 34.6317 | 30.2973 |  |  |  |  |
| 8.15348 | 4.03432 |  |  |  |  | 24.1306 | 14.7134 | 37.7678 | 30.3641 |  |  |  |  |
| 8.54801 | 5.77667 |  |  |  |  | 24.7367 | 14.9567 | 37.9793 | 31.6275 |  |  |  |  |
| 8.81218 | 6.17411 |  |  |  |  | 28.0594 | 20.3237 | 39.9704 | 24.2149 |  |  |  |  |
| 9.24754 | 2.6078 |  |  |  |  | 18.8580 | 22.1352 | 24.0153 | 18.4965 |  |  |  |  |
| 9.4979 | 6.70416 |  |  |  |  | 30.9358 | 20.3744 | 24.9602 | 22.3463 |  |  |  |  |
| 9.80834 | 6.26115 |  |  |  |  | 34.8027 | 22.2047 | 26.7496 | 21.0534 |  |  |  |  |
|  |  |  |  |  |  | 35.4383 | 22.3381 | 27.686 | 19.3139 |  |  |  |  |
|  |  |  |  |  |  | 18.6008 | 22.5822 | 31.2618 | 22.1074 |  |  |  |  |
|  |  |  |  |  |  | 19.8401 | 16.1731 | 37.6123 | 28.7228 |  |  |  |  |
|  |  |  |  |  |  |  |  | 44.4036 | 29.4406 |  |  |  |  |
|  |  |  |  |  |  |  |  | 53.7051 | 31.8304 |  |  |  |  |

**Notes.**

OTM = Olive tail moment, TailDNA%= % DNA in the comet tail; 0 mg/mL concentration of Gelsemine was used as Blank control, and DMSO concentration (0.1 %) was used as Negative control (NC).


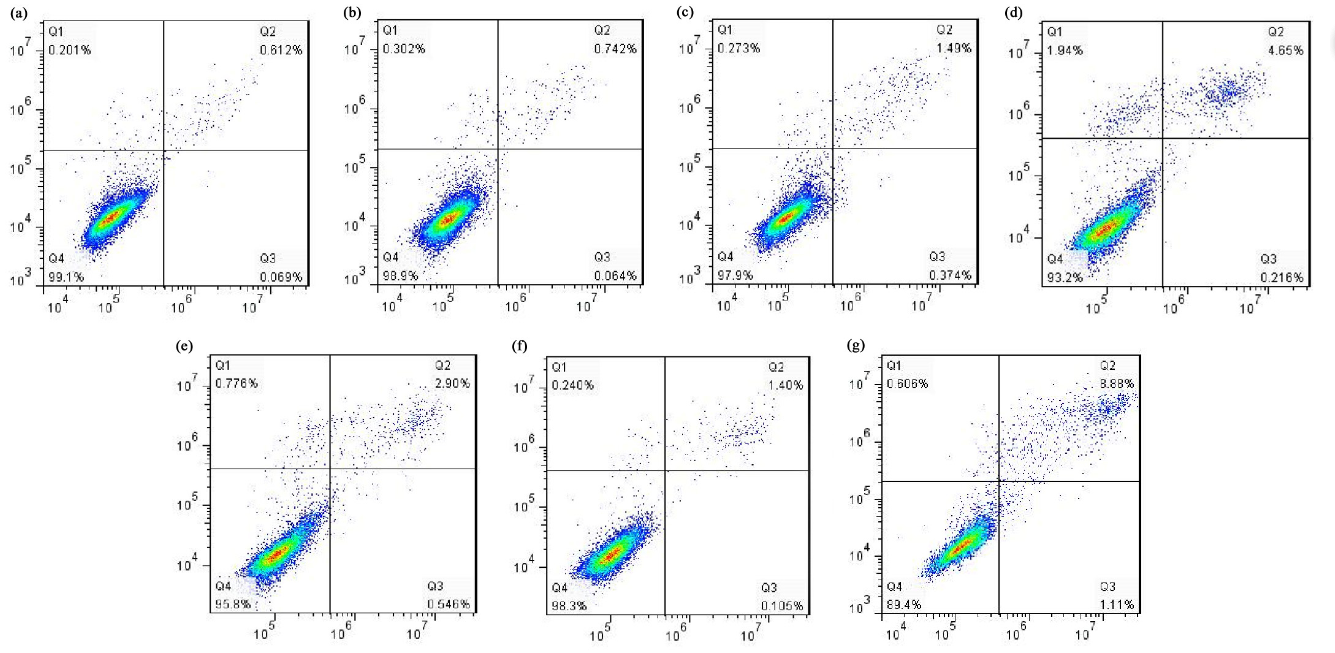


**Supplementary figure S1** Diagram of the detection of apoptosis in *T. thermophila* using flow cytometry. (a) blank control; (b) negative control (NC); (c) 0.05 mg/mL; (d) 0.1 mg/mL; (e) 0.2 mg/mL; (f) 0.4 mg/mL; (g) 0.8 mg/mL. Q1: the cells in this region are necrotic cells; Q2: the cells in this region are late apoptotic cells; Q3: the cells in this region are early apoptotic cells; Q4: the cells in this region are living cells.


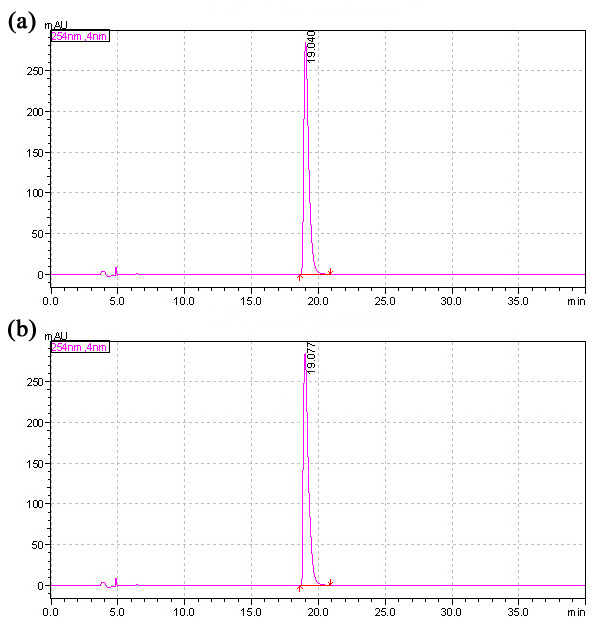


**Supplementary figure S2** The liquid chromatogram of Gelsemine exposed to ultraviolet germicidal irradiation (103 μW/cm^2^) for 1 h prior to the experiment. (a) Pre-irradiation; (b) After irradiation.


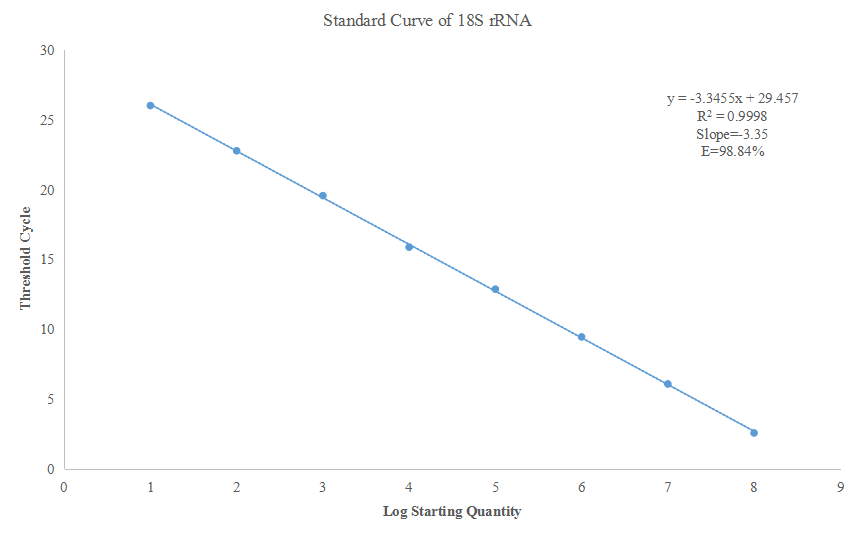


**Supplementary figure S3** The standard curve of *18S rRNA* using in the qRT-PCR. Correlation coefficient (R2), Slope (S) and PCR efficiency (E). R^2^=0.9998, S=3.35, and E=98.84%.


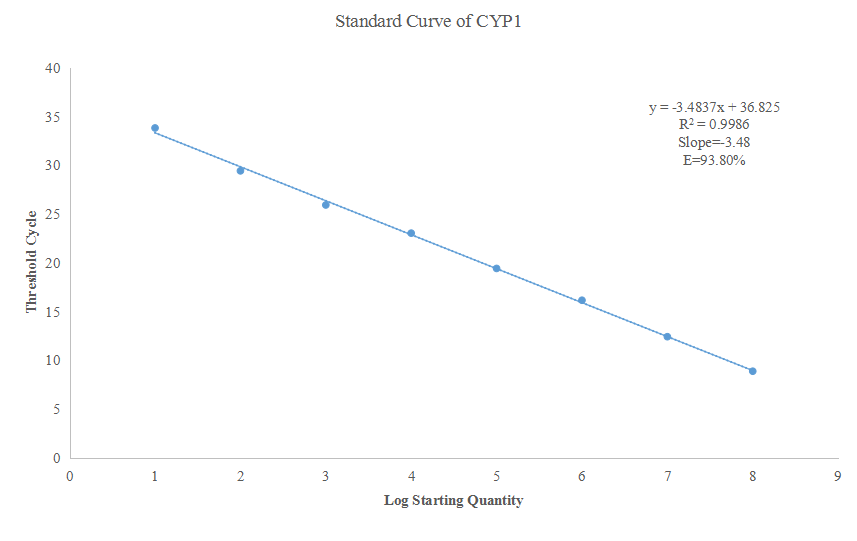


**Supplementary figure S4** The standard curve of *CYP1* using in the qRT-PCR. Correlation coefficient (R2), Slope (S) and PCR efficiency (E). R^2^=0.9986, S=3.48, and E=93.80%.


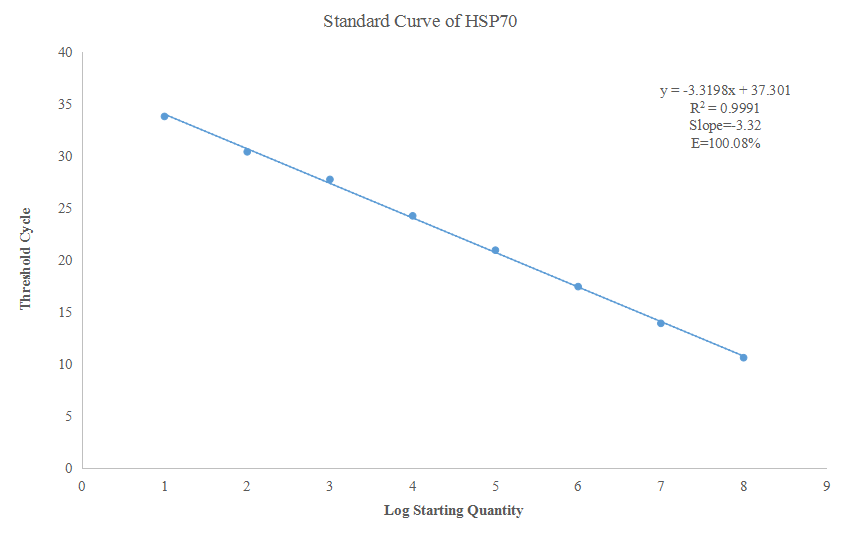


**Supplementary figure S5** The standard curve of *HSP70* using in the qRT-PCR. Correlation coefficient (R2), Slope (S) and PCR efficiency (E). R^2^=0.9991, S=3.32, and E=100.08%.


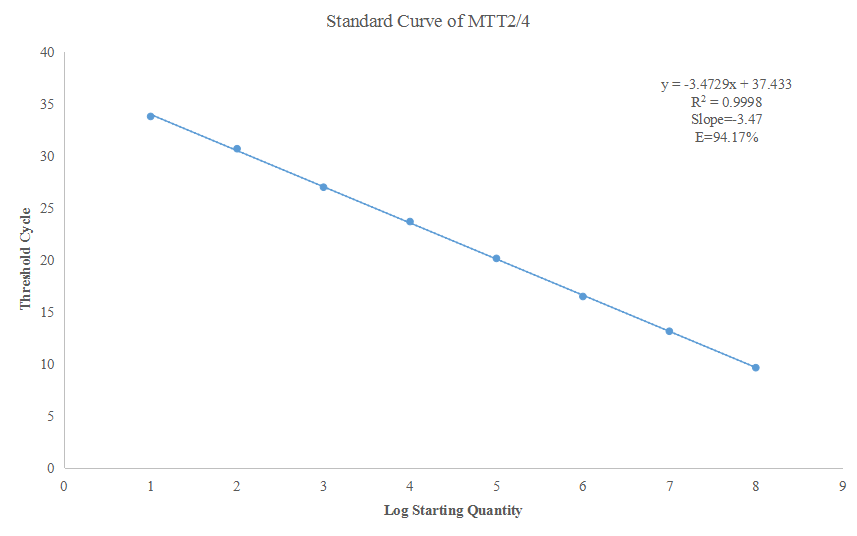


**Supplementary figure S6** The standard curve of *MTT2/4* using in the qRT-PCR. Correlation coefficient (R2), Slope (S) and PCR efficiency (E). R^2^=0.9998, S=3.47, and E=94.17%.


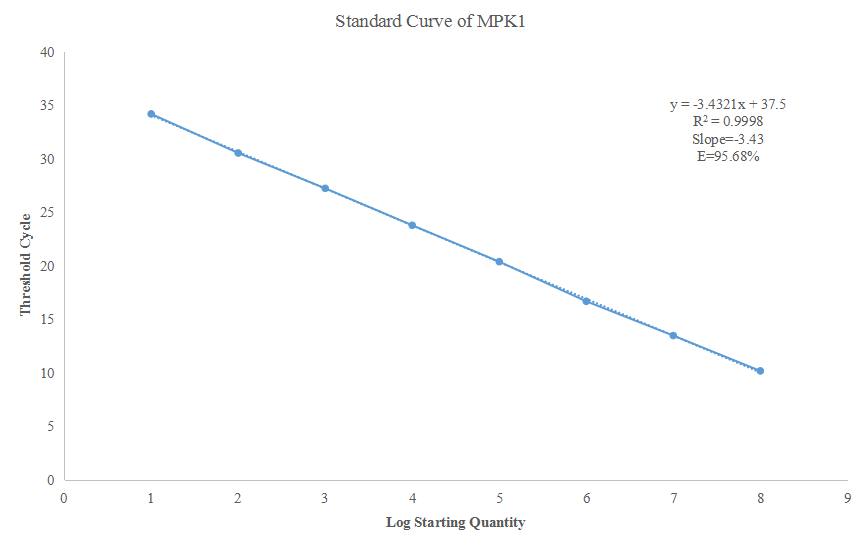


**Supplementary figure S7** The standard curve of *MPK1* using in the qRT-PCR. Correlation coefficient (R2), Slope (S) and PCR efficiency (E). R^2^=0.9998, S=3.43, and E=95.68%.


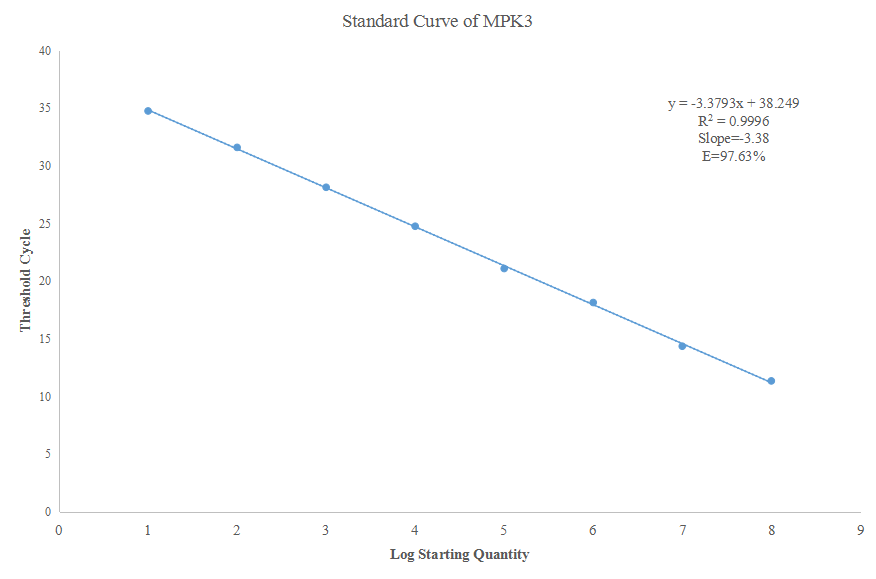


**Supplementary figure S8** The standard curve of *MPK3* using in the qRT-PCR. Correlation coefficient (R2), Slope (S) and PCR efficiency (E). R^2^=0.9996, S=3.38, and E=97.63%.


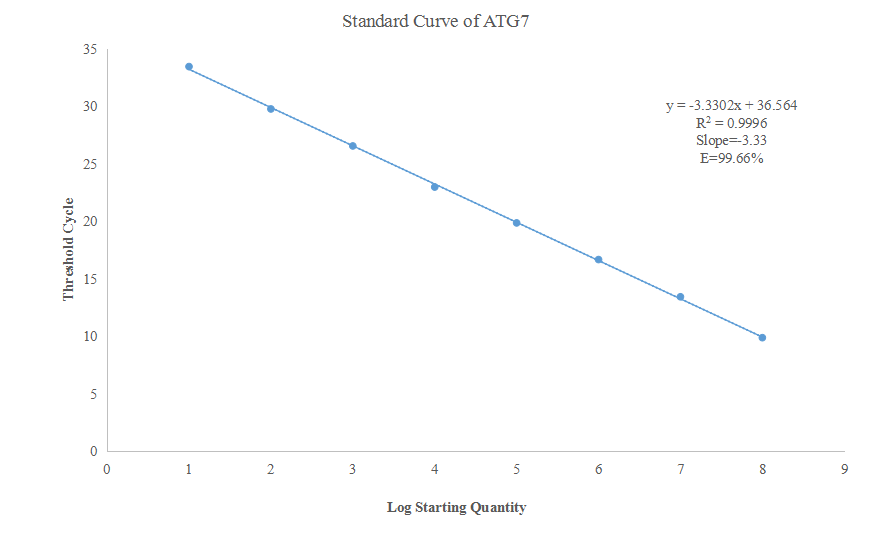


**Supplementary figure S9** The standard curve of *ATG7* using in the qRT-PCR. Correlation coefficient (R2), Slope (S) and PCR efficiency (E). R^2^=0.9996, S=3.33, and E=99.66%.
